# DNA barcoding as new diagnostic tool to lethal plant poisoning in herbivorous mammals

**DOI:** 10.1371/journal.pone.0292275

**Published:** 2023-11-15

**Authors:** Sandra Schweikle, Annette Häser, Sascha Wetters, Moses Raisin, Maica Greiner, Kerstin Rigbers, Ulrike Fischer, Klaus Pietsch, Michael Suntz, Peter Nick

**Affiliations:** 1 Molecular Cell Biology, Botanical Institute, Karlsruhe Institute of Technology, Karlsruhe, Germany; 2 State Institute for Chemical and Veterinary Analysis Karlsruhe, Karlsruhe, Germany; 3 State Institute for Chemical and Veterinary Analysis Freiburg, Freiburg im Breisgau, Germany; Institute for Biological Research, University of Belgrade, SERBIA

## Abstract

Reliable identification of plant species in the digestive tract of a deceased animal often represents the major key to diagnose a lethal intoxication with poisonous plants in veterinary pathology. In many cases, identification of the species is challenging or even impossible because the diagnostic morphological features have been degraded, and because the interpretation of such features requires a considerable expertise in plant anatomy and biodiversity. The use of DNA barcoding markers can support or even replace classical morphological assessment. While these markers have been widely used for plant taxonomy, their forensic application to clarify causes of animal poisoning is novel. In addition, we use specific single-nucleotide polymorphisms as fingerprints. This allows for a clear decision even in cases, where the conventionally used statistical e-values remain ambiguous. In the current work, we explore the feasibility of this strategy in a couple of exemplary cases, either in concert with anatomical diagnostics, or in cases where visual species identification is not possible, or where chemical toxin detection methods are not well established, complex, time consuming and expensive.

## 1. Introduction

Animal poisoning by plants represents something like the “dark matter” of veterinary forensics. Since there is no common European database and not even any legislation requiring to report such cases, the evidence is mostly anecdotical and not systematic. Several studies conducted in retrospect, have revealed, however, that animal intoxication by plants is not a rare phenomenon. A systematic study for Germany estimates that around 20% of cases are linked with plant poisoning (MacFarland *et al*., 2017) [1], which is in line with a series of similar studies on different groups of animals conducted on the European level (Berny et al., 2019 [2]; Guitart et al. 2010) [3]. If ruminants, equines, and rodents are considered, plants account even for the majority of fatal cases (MacFarland *et al*., 2017) [1]. Acute plant poisonings cause unexpected death of otherwise healthy animals. The sudden acuity of symptoms often raises the question of criminal acts, such as malicious cruelty against animals.

The real incidence of plant-caused poisonings might be even higher because its diagnosis is far from trivial. Sometimes, the combination of necropsy data and morphological identification of plant parts in the gastrointestinal track allows to come up with a hypothesis on the cause of the acute death. In fact, this has been successful for a couple of pertinent plants, such as *Nerium oleander* (Langford and Boor, 1996) [4], *Senecio vulgare* (Moyano *et al*., 2006) [5], or alkaloid containing Solanaceae such as *Datura stramonium* (Bofill *et al*., 2007) [6]. However, the majority of cases may remain undetected. For instance, almost ¾ of poisonings of sheep and goats in Greece could not be clarified (Antoniou *et al*., 2019) [7]. Chemical analysis of the toxin itself is usually not available under the conditions of routine and forensic diagnostic veterinary pathology due to a lack of laboratory capacities. To identify the remnants of an ingested plant would be challenging even for experts of plant taxonomy, since informative plant structures were often destroyed by the digestive activity of the still physiologic gut content. Therefore, a rapid and robust method of identification is needed not only to clarify unclear cases of sudden animal death, but also to get insight into the epidemiological aspects of this phenomenon.

The DNA of an organism does not change, no matter, which tissue is investigated, nor which developmental state is addressed, nor, which environmental conditions prevailed, before the plant was ingested by the herbivore. Based on differences in the sequence of specific markers, it is possible to genetically barcode a taxon of interest and to discern it from similar taxa. By the joint efforts of molecular taxonomists all over the planet, a set of such genetic barcodes has been established that meet three criteria: they are universal, but at the same time sufficiently variable between different taxa, and amenable to reliable sequencing (CBOL, 2009) [8]. Mitochondrial barcodes prevalent in animal molecular taxonomy, are not suited for plants, because they do not change with sufficient velocity. Instead plastidic markers, such as the coding regions of the genes *1*,*5-ribulose bisphosphate carboxylase Large subunit* (*rbcL*) or *maturase K* (*matK*), or intergenic spacers such as the *trnH-psbA* are commonly used along with the two *internal transcribed spacers* (*ITS1* and *ITS2*) regions of the rRNA genes as nuclear markers. Genetic barcoding is more challenging in plants as compared to animals, because the so-called taxon gap between neighbouring species is often smaller than the genetic variation within these species (Fazekas *et al*., 2009) [9]. Nevertheless, DNA barcoding performs well in applications, where taxonomical identity of processed or otherwise deformed specimens is relevant. Examples are applications targeted to the traceability (for review see Galimberti *et al*., 2013) [10], or authentication of commercial herbal products. A systematic metastudy (Ichim, 2019) [11] revealed that on a global scale around half of the products were adulterated, giving evidence for the efficiency of this authentication strategy.

In fact, the potential of genetic barcoding for forensic applications is attracting progressive interest. To prepare future application, sequence catalogues have been prepared for poisonous plants that are relevant for human intoxications (Wang *et al*., 2021 [12]; Nithaniyal *et al*., 2021) [13]. However, to the best of our knowledge, the transfer to real-world applications is missing so far. In the current work, we want to fill this gap.

It should be emphasised that this work is a forensic study, which means that it deals with individual real-world cases of lethal poisoning from South Germany, not animals that have been sampled statistically from a laboratory study. The circumstances, how these animals were raised or how they died, are usually not known. They were found dead by their owners and brought to the routine veterinary inspection. The storyline, therefore, deviates from a conventional animal study. Due to the nature of the objects analysed here, statistical treatment is not possible. Instead, we structure this study along a gradient of challenge. This gradient begins with situations, where gut content and circumstantial evidence still provide certain morphological and anatomical cues that can help to identify the causative plant, towards situations, where such cues have been completely eliminated due to digestive activity. Barcoding has been successfully used in ecological studies (Valentini *et al*., 2009) [14] also to characterise the diet of animals (Kartzinel *et al*., 2015) [15]. However, to the best of our knowledge, this study represents the first case, where barcoding has been employed in veterinary forensics. We show that the search for diagnostic fingerprint in the sequence allows for better resolution as compared to a global assignment of inferred identity based on the global e-value of the target sequence from a BLAST search in public databases, we explore the limitations of this methodology, and we come up with recommendations to unfold the full forensic potential of this strategy.

## 2. Materials and methods

### 2.1. Reference plants

The samples to be identified came from the routine active of the State Institute of Chemical and Veterinary Analysis in Freiburg and Karlsruhe. The anatomical features as well as the generated barcoding sequences were compared to those from the respective reference plants from the collection of the Botanical Garden of the Karlsruhe Institute of Technology. Details and voucher numbers are listed in Table 1.

**Table 1:** Reference plant material used in this research; scientific name; common name; ID of the voucher specimen cultivated in the Botanical Garden of the KIT; GenBank accession numbers of the sequences generated during this study.

Identity	Common name	KIT ID	marker	GenBank
*Camellia japonica* L.	Japanese camelia	5477	*trnH-psbA*	ON542504
*Camellia sinensis* (L.) Kuntze	tea plant	7780	*trnH-psbA*	ON542505
*Nerium oleander* L.	rose bay	9450	*ycf1b*	ON722358
*Nerium oleander* L.	rose bay	9450	*trnH-psbA*	ON603329
*Nerium oleander* L.	rose bay	1890	*ycf1b*	ON722357
*Nerium oleander* L.	rose bay	1890	*trnH-psbA*	ON603328
*Prunus laurocerasus* L.	cherry laurel	9451	*trnH-psbA*	
*Picea abies* L.	common spruce	collected	*trnH-psbA*	OP012852
*Pieris japonica* Thunberg L.	Japanese Andromeda	5488	*trnH-psbA*	OP012855
*Robinia pseudoacacia* L.	black locust	collected 1	*ITS 1*	ON929301
*Robinia pseudoacacia* L.	black locust	collected 2	*ITS 1*	ON929302
*Trifolium pratense* L.	white clover	collected	*ITS 1*	OP012864
*Poa trivialis* L.	rough bluegrass	collected	*rcbL*	OP013000
*Fagus sylvatica* L.	common beech	collected	*trnH-psbA*	OP012858

The taxonomic identities of the reference plants were verified according to taxonomic keys of the Flora of China (*Camellia*, *Pieris)* [16], and Aichele and Schwegler (1994, other taxa) [17].

### 2.2. Anatomical analysis

Fresh leaves or gut content were first inspected by stereo microscopy (S6D, Leica, Bensheim, Germany), with focus on leaf veins, crystals, and other specific structures. To visualise cellular details, tangential hand sections were depigmented with 60% chloral hydrate (Carl Roth, Karlsruhe, Germany) upon heating up above the flame of a Bunsen burner. The specimens were analysed by bright-field microscopy (DM750, Leica, Bensheim), sometimes under polarised light. All images were documented by a digital system (EC3, Leica, Bensheim).

### 2.3. Extraction of DNA

Genomic DNA was extracted with cetyl trimethyl ammonium bromide (CTAB) according to Doyle and Doyle (1987) from 100 mg of shock-frozen and homogenised plant material (TissueLyzer, Qiagen, Hilden, Germany). After incubation in 1 ml of 1.5% w/v CTAB for 1 h at 65°C, the samples were mixed with 630 μl of chloroform/isoamylalcohol (24:1), shaken horizontally for 15 minutes, and spun down for ten minutes at 17,000 g. The upper aqueous phase, containing the DNA, was transferred into a fresh 2 mL reaction tube and the DNA precipitated with 2/3 v/v of ice-cold isopropanol. The DNA was sedimented by centrifugation (10 min, 17,000 g, 4°C), the sediment washed with 1 mL 70% EtOH, and the EtOH removed by drying in a vacuum centrifuge for 15 minutes, and the DNA precipitate finally dissolved in 50 μL nuclease-free H_2_O (Lonza, Biozym) containing 5 μg RNAse A (Qiagen, Hilden, Germany). The concentration and purity of the eluted DNA was determined spectrophotometrically (NanoDrop ND-100, peqlab).

### 2.4. Genomic PCR for barcoding markers

The different barcoding markers were amplified from 75 ng template DNA in a reaction volume of 30 μL with 3 μL 10 x reaction buffer (Thermopol, New England Biolabs), 3 μL of Bovine Serum Albumine (10 mg^.^mL^-1^), 0.6 μL of dNTPs (200 μM, New England Biolabas), 0.6 μL0 of both oligonucleotide primers (Merck, Darmstadt, see Table 2), and 0.3 μL of Taq polymerase (5 U, New England Biolabs).

**Table 2:** Oligonucleotid primers used for amplifying the genetic barcodes used in this study.

target	sequence	reference
*psbA-trnH*	fw 5’-GTTATGCATGAACGTAATGCTC-3’	Sang *et al*. (1997) [18]
	rev 5’-CGCGCATGGTGGATTCACAATCC-3’	Tate and Simpson (2003) [19]
*ycf1b*	fw 5’-TCTCGACGAAAATCAGATTGTTGTGAAT-3’	Dong *et al*. (2015) [20]
	rev 5’-ATACATGTCAAAGTGATGATGGAAAA-3’	
*ITSA*, *B*	fw 5’-GGAAGGAGAAGTCGTAACAAG-3’	Chiou *et al*. (2007) [21]
	rev 5’-CTTTTCCTCCGCTTATTGATATG-3’	
*rbcL*	fw 5’- ATGTCACCACAAACAGAGACTAAAGC -3’	Kress and Erickson (2007) [22]
	rev 5’- CGTGGTGGACTTGATTTTAC -3’	

Amplification was conducted with an initial denaturation step of 120 s at 95°C followed by 35 cycles of denaturation at 95°C for 30 sec (45 sec in case of *trnH-psbA*), annealing at 60° for 30 sec, synthesis at 68° for 60 s, and a final elongation at 68° for 300 s. Amplicons were evaluated by gel electrophoresis using NEEO ultra-quality agarose (Carl Roth, Karlsruhe, Germany). DNA was visualised using Midori Green (NIPPON Genetics EUROPE, Germany) under blue light excitation. The fragment sizes of the amplicons were determined by a 100 bp size standard (New England Biolabs). Prior to being sent out for sequencing (Eurofins, Konstanz, Germany), the amplified DNA was purified using the protocol of the MSB Spin PCRapace Kit (Stratec). The obtained sequences were deposited in the NCBI database (for the accession numbers of the reference plants see Table 1, for those of the gut samples see Table 3).

**Table 3:** List of sequences amplified from the gut samples.

animal	common name	ID	marker	GenBank
*Vicuna pacos* L.	alpaka	CVUA_FR 1	*trnH-psbA igs*	ON542503
*Vicuna pacos* L.	alpaka	CVUA_FR 2	*trnH-psbA igs*	ON603327
*Vicuna pacos* L.	alpaka	CVUA_FR 2	*ycf1b*	ON722356
*Capra aegagrus hircus* L.	goat	CVUA_FR 1285	*trnH-psbA igs*	OP012851
*Bos taurus* L.	cattle	CVUA _FR 4_1	*its*	ON929303
*Bos taurus* L.	cattle	CVUA _FR 4_2	*Its*	ON929304
*Equus caballus* L.	horse	CVUA_KA A570_A	*rcbL*	OP012859
*Equus caballus* L.	horse	CVUA_KA A570_B	*rcbL*	OP012860
*Equus caballus* L.	horse	CVUA_KA A570_C	*rcbL*	OP012861
*Equus caballus* L.	horse	CVUA_KA A570_D	*rcbL*	OP012862
*Equus caballus* L.	horse	CVUA_KA A570_E	*rcbL*	OP012863
*Equus caballus* L.	horse	CVUA_KA A570_its_1	*its 1*	OP012996
*Equus caballus* L.	horse	CVUA_KA A570_its_2	*its 1*	OP012997
*Equus caballus* L.	horse	CVUA_KA A570_its_3	*its 1*	OP012998
*Equus caballus* L.	horse	CVUA_KA A570_its_4	*its 1*	OP012999
*Equus caballus* L.	horse	CVUA_KA A570_1	*trnH-psbA*	OP012856
*Equus caballus* L.	horse	CVUA_KA A570_2	*trnH-psbA*	OP012857

### 2.5. Sequence analysis

Sequence reads were generated in both directions and aligned using the Muscle algorithm of the software package MEGA7 (https://www.megasoftware.net/). Discrepancies in these alignments were then checked in the respective chromatogramm and edited manually. The edited sequences were then used as input for a BLAST search in GenBank to the NCBI browser and sequences from the respective taxa, their closest relatives, and appropriate outgroups were collected by means of the Taxonomy View routine. The sequences from the animal samples were aligned with those from the respective reference plant, and the sequences obtained from GenBank and the respective phylogenetic trees were inferred using the Neighbour-Joining algorithm and were visualised using the Tree Explorer integrated into MEGA. Informative SNPs or indels were counted for all available sequences of the respective taxa to estimate the taxonomic support for the respective polymorphism.

## 3. Results

### 3.1. Morphological diagnostics of animal poisonings is limited

**Fig 1:**
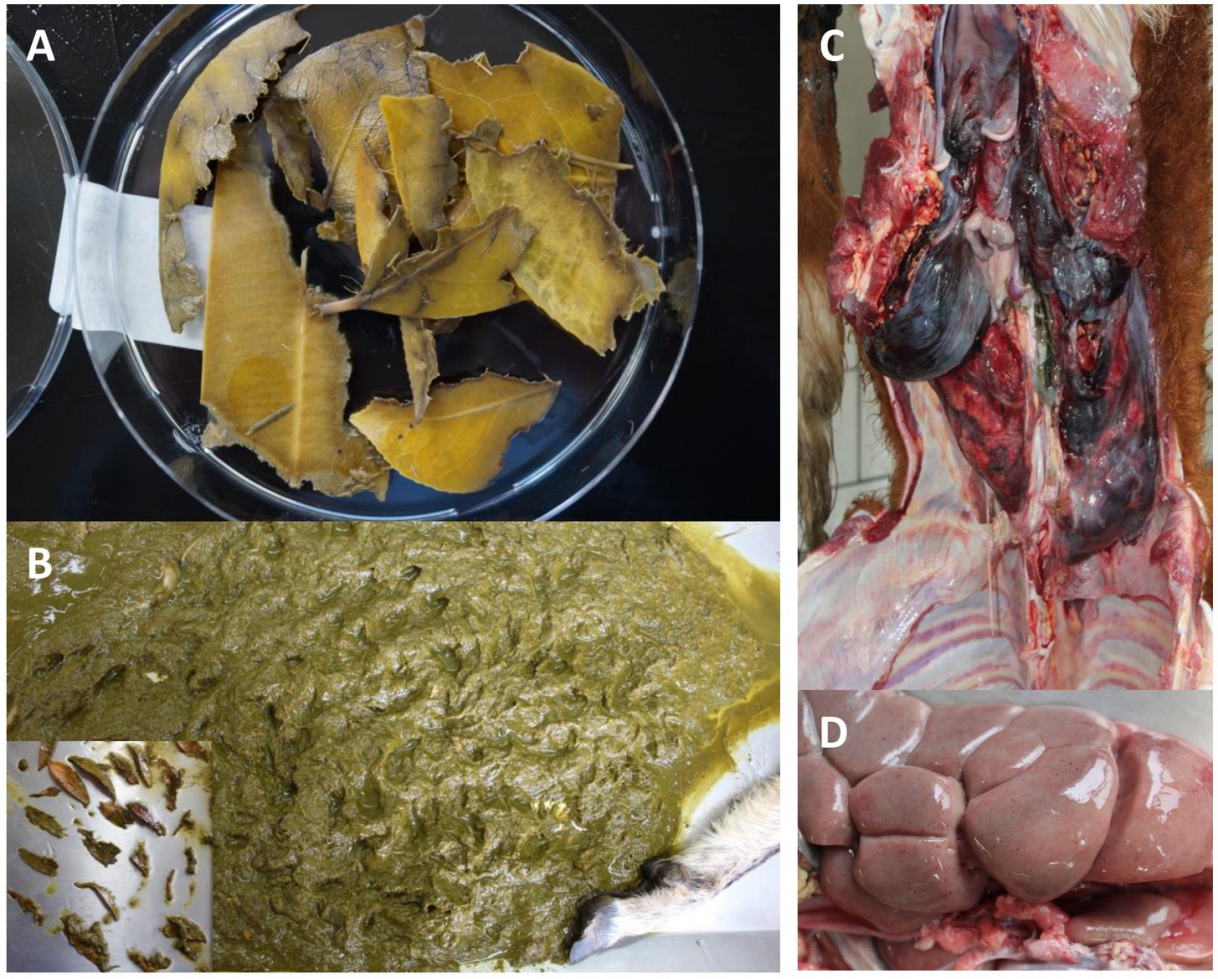
Representative aspects of plant-based animal poisoning with respect to anatomical preservation (**A, B**) and symptoms (**C, D**). **A** Well-preserved leaf mixture (*Nerium oleander*, *Camellia japonica*) from a dead alpaca (case 1). **B** Homogenously ground ruminal filling from a goat (case 4) with few residual nests of masticated, unidentifiable leaves (insert). **C** Massive acute perirenal hemorrhage and edema in cattle (case 5). **D** Detail from the same case showing renal degeneration with petechiae.

The study is based on a collation of cases from veterinary routine (Fig 1) that are arranged in a gradient with respect to preservation of morphological traits still detectable in the gut content. This gradient allows to test out the limitations of microscopic diagnostics and to breach these limitations by means of DNA barcoding markers. In total, six carcasses from four different herbivorous species were used for this study. These were submitted to a diagnostic department of a state veterinary institute by the owners. The cause of death was mostly unclear, and routine veterinary diagnostic procedures such as necropsy, histology and microbiology did not suffice to determine the cause of death. We, therefore, analysed stomach or forestomach content by DNA barcoding and extended the resolution of this approach by integrating authenticated reference plants along with specific Single-Nucleotide Polymorphisms (SNPs) and indels corrobated by comparisons with publicly available sequences from the respective plant species. In none of the cases the death had been acute without any preceding history of illness or specific symptoms that were anamnesically reported by the clinician or owner. All of the deceased animals had been fed and kept under conventional and inconspicuous barnyard conditions (outdoor and stable).

In a few cases, remnants found in the animal gut are sufficiently preserved to distinguish taxonomically relevant traits, such that it is possible to infer the origin of these remnants, as exemplified by the case of an alpaca that had eaten leaves from *Camellia japonica* (not in sufficient quantity to explain the death) and of *Nerium oleander*, which is sufficiently toxic to account for the death of the animal (Fig 1A). However, in many cases, the digestion processes have homogenised the plant material to a degree that even an experienced plant anatomist cannot recognise anything of value for taxonomical identification, as shown for the ruminal fillings of a deceased goat (Fig 1B). While further forensic evidence can be collected from inspecting the inner organs of the animal, which often allows to pinpoint the organ failure responsible for the death, this does often not help to pinpoint the toxic plant that has caused this organ failure. For instance, the detection of a perirenal hemorrhage and edema in cattle (Fig 1C), accompanied by renal degeneration with petechiae (Fig 1D), provide evidence that this animal experienced a breakdown of kidney function, which certainly can explain its death, but does not give any information whatsoever on the primary cause for the renal collapse. We decided, therefore, to arrange the cases along a gradient (Table 4).

**Table 4:** Survey of the cases of animal deaths caused by plant intoxication addressed in the current study. The list is according to progressive loss of anatomical features available for morphological identification of the ingested plant material.

case	species	case history	main pathomorphological findings/diagnoses	inferred poisonous plant
1	alpaca *Vicugna pacos*	ran away, peracute death	acute cardiovascular failure (shock) with acute parenchymal congestion and lung edema, forestomachs filled with homogenously ground plant material and a few non-identifiable, masticated leaves (suspected to be Oleander and others)	oleander (*Nerium oleander*)
2	goat *Capra aegagrus hircus*	not known	advanced state of decomposition, catarrhal enteritis, ruminal filling: homogenously ground plant material and few non-identifiable, masticated leaves (suspected to be *Azalea* or *Prunus*)	cherry laurel (*Prunus laurocerasus*)
3	goat *Capra aegagrus hircus*	shock, tumbling down	acute cardiovascular failure (shock) with acute parenchymal congestion and lung edema, brain edema, rumen massively filled with needles and branches of conifere material, smell of essential conifere oil	common spruce (*Picea abies*)
4	goat *Capra aegagrus hircus*	yelling, weakness, oral froth	acute cardiovascular failure (shock) with acute parenchymal congestion and lung edema, ruminal filling: homogenous plant material with fragments from non-identifiable leaves (suspected to be *Azalea* or *Prunus*)	Chinese *Pieris* x Japanese Andromeda *Pieris formosa x japonica*
5	cattle *Bos taurus*	peracute death	uremia, massive acute perirenal edema and hemorrhage, massive acute diffuse renal tubular degeneration, acute parenchymal congestion, and lung edema, homogenously ground, non-identifiable, plant material in rumen	black locust *Robinia pseudoacacia*
6	Horse *Equus caballus*	severe increased muscle and liver lactate values; immobility; overflow incontinence; loss of deep sensibility	Moderate myopathy of heart, neck, diaphragm and extremeties.	beech *Fagus sylvatica*

### 3.2. *Dosis sola venenum facit*: *Camellia* versus *Nerium*

**Fig 2:**
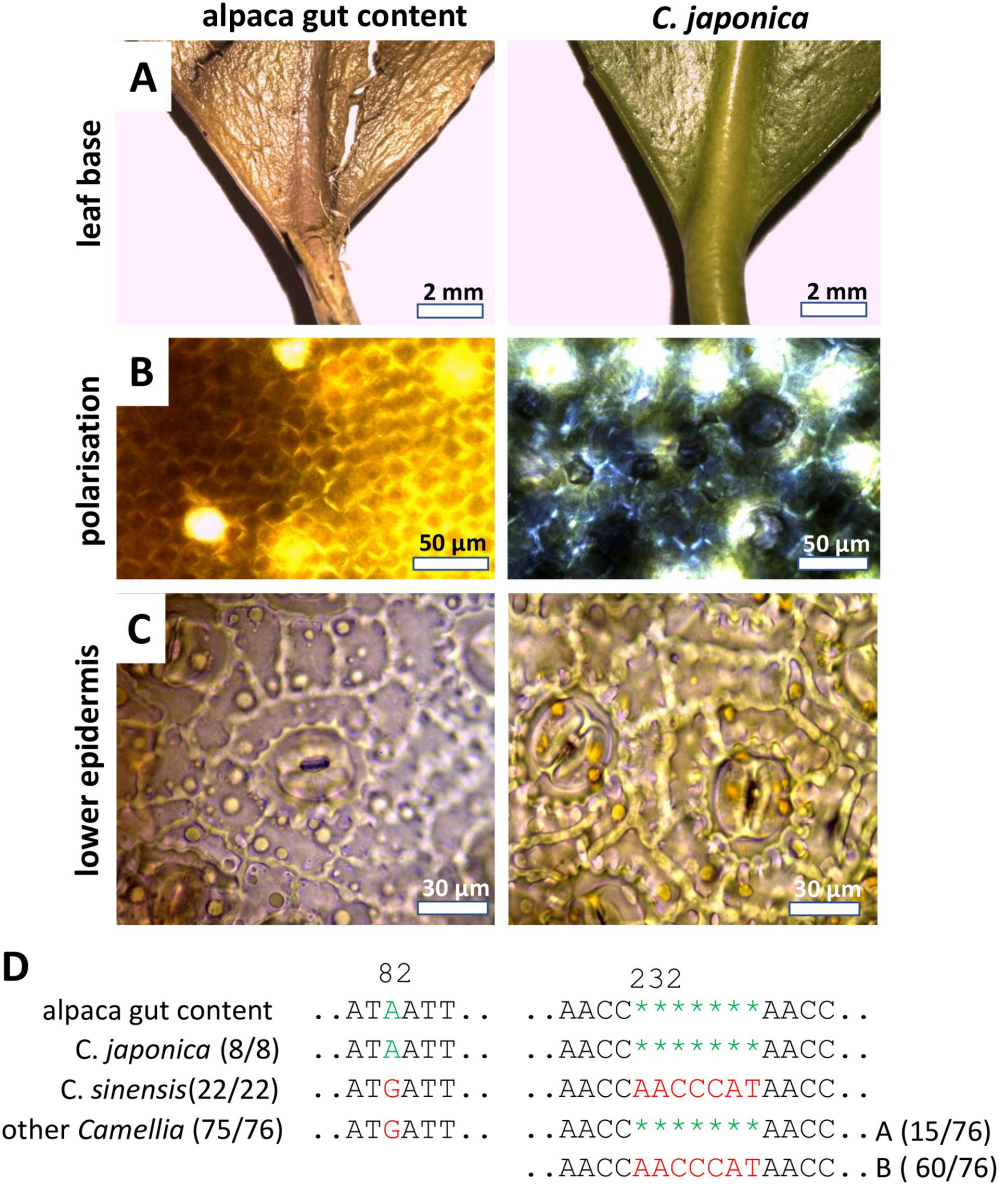
Morphological and anatomical comparison between macerated leaf fragments from the stomach of alpaca (case 1) and a reference plant for *Camellia japonica* from the KIT Botanical Garden. **A** leaf base with petiole. **B** crystal druse visualised by polarisation microscopy. **C** Anisocytic stomata from the lower epidermis. **D** diagnostic fingerprints in the *trnH-psbA* marker.

The first case was a deceased alpaca that had died with cardiovascular failure and shock symptoms linked with a pulmonary edema (Table 4). The forestomachs of this animal were filled with homogenised plant material (obviously already ingested some time ago) and some leaf fragments that were subjected to microscopical diagnostics. By their slender leaf base and venation patterns (Fig 2A), birefringent crystals in the mesophyll (Fig 2B), and anisocytic stomatal complexes (Fig 2C), these fragments were suspected to originate from a *Camellia* species and, in fact, the resemblance with corresponding samples from an authenticated reference plant for *C*. *japonica* from the collection of the Botanical Garden of the KIT was striking (compare Fig 2A–2C, left and right-hand columns). To test this assumption, we purified genomic DNA from the gut content and amplified the plastidic marker *trnH-psbA*. Based on the alignment of the sequence recovered from the alpaca, reference plants for the suspected *C*. *japonica* and the morphologically similar *C*. *sinensis*, and all sequence homologues we could recover from GenBank along with *Apterosperma oblata* as outgroup (S1 Data), we inferred a phylogenetic tree using the distance-based and robust Neighbour Joining algorithm (S1 Fig). Here, the sequence from the gut content and the reference plant for *C*. *japonica* were found to map to the same location. However, different species from the genus *Camellia* were not separated into different clades, but were interspersed, such that the statistical support for to exclude the possibility that the gut sample contained *C*. *sinensis* (or other species of *Camellia*) rather than *C*. *japonica* was lacking. Rather than using overall sequence similarity as criterion, we were searching for species specific polymorphisms in the sequence. In fact, we were able to spot a specific A in position 82 of the alignment, which was found in the sequence from the gut, in the reference plant of *C*. *japonica* and in all 7 GenBank sequences declared as *C*. *japonica* (Fig 2D). This A was replaced by a G in all 22 accessible sequences for *C*. *sinensis*, and in 75 from 76 sequences recovered for other members of the genus. Furthermore, a gap of 7 nucleotides was found at position 232 of the alignment in the gut sample as well as in all *C*. *japonica* sequences. This gap was missing in all sequences from *C*. *sinensis*. It was also missing in the majority (60 from 76) sequences from other *Camellia* species however, it was present in a minor group of these sequences. Thus, the combination of a Single Nucleotide Polymorphism (SNP) in position 82 and the presence of the 7-bp deletion in position 232 is a specific fingerprint that separates *C*. *japonica* from all other *Camellia* species, where this marker was available. The fact that both features were seen in the gut sample, is compelling evidence that the alpaca had ingested leaves from *C*. *japonica*. However, since Camelia is not known to be toxic (*C*. *sinensis* is even used as beverage in many cultures), we returned to the gut content with more scrutiny.

**Fig 3:**
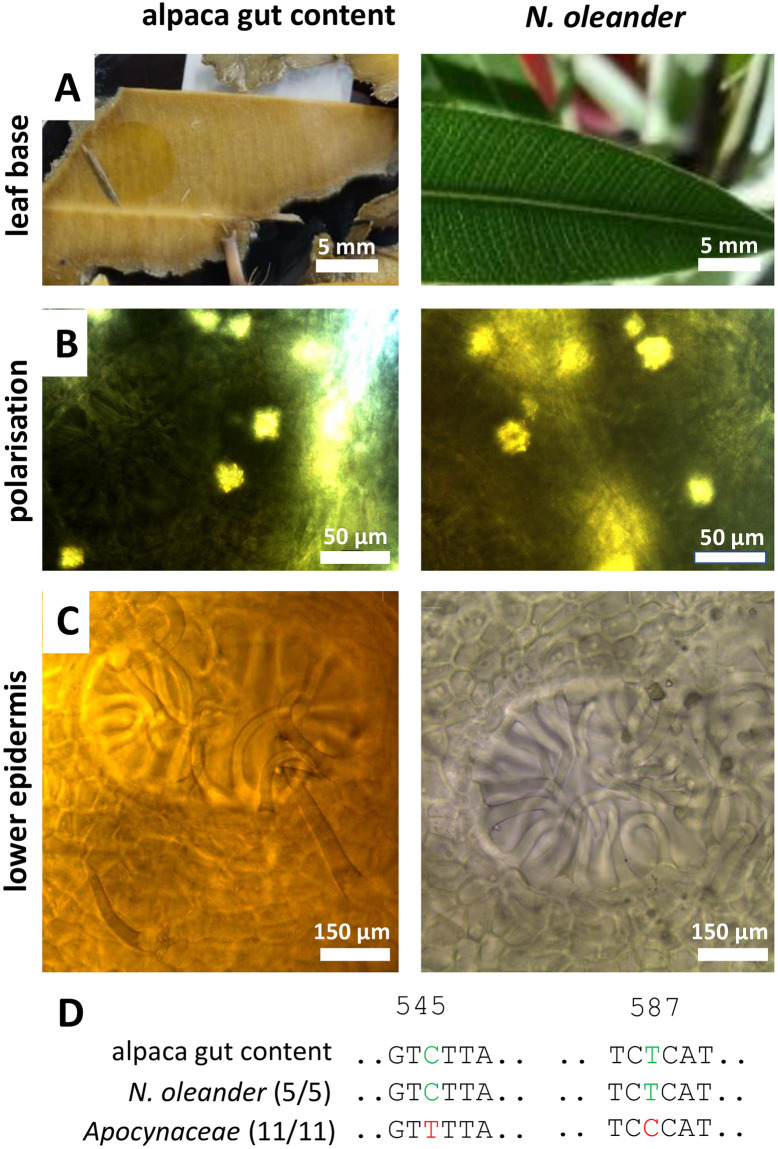
Morphological and anatomical comparison between macerated leaf fragments from the stomach of alpaca (case 1) and a reference plant for *Nerium oleander* from the KIT Botanical Garden. **A** lamina with characteristic parallelilty of lateral veins **B** crystal druse visualised by polarisation microscopy. **C** characteristic concave stomata with trichomes in the lower epidermis. **D** diagnostic fingerprints in the *ycf1b* marker.

In fact, we recovered from the gut content a few fragments of a different type of leaf, which by its lanceolate shape and conspicuous venation (Fig 3A) was suspected to originate from Rose Bay (*Nerium oleander*). Upon microscopic diagnostics, birefringent crystals were detected by polarisation microscopy (Fig 3B), and characteristic concave stomata covered by trichomes (Fig 3C) further corrobated this working hypothesis. The attempt, to identify the plants by the *trnH-psbA* marker were not successful though. The alignment (S2 Data) revealed numerous insertions in the alpaca sequence that were absent from all *Nerium* sequences as well as relatives from the same family. On the other hand, the regions interspersed between these gaps were not very informative. As a result, while the sequences from our reference plants clustered well with the sequences deposited in GenBank (S2A Fig), the alpaca sequence showed an odd location, almost as distant as the outgroup, *Gentiana pannonica*.

Since the *trnH-psbA igs* did not help here, we tested the *ycf1b* marker, a plastidic gene, which is essential for photosynthesis, such that alignments are not affected by gaps and insertions, while on the other hand, several variable regions allow for good discriminative power (Dong *et al*., 2015) [20]. In fact, this marker produced a more solid alignment (S3 Data), and the phylogenetic tree inferred from this alignment placed the alpaca sequence close to the reference plants and database sequences for *N*. *oleander*, while the other genera from the same family (Apocynaceae) were clearly clustering separately (S2B Fig). We were able to detect two SNPs, a C at position 545 shared between the sequence recovered from the alpaca gut and all five *N*. *oleander* sequences, while all other (11) homologues from other members of the Apocynaceae showed a T at this position (Fig 3D). Likewise, a T at position 587 was separated the sequence from the gut content and all N. oleander sequences from their Apocynaceae counterparts that harboured a C at this position. Thus, two very specific fingerprints that were only found in *N*. *oleander* provided compelling evidence that the alpaca had eaten rose bay before its death. Due to the steroid glycosides, such as oleandrin, even small amounts of rose bay are toxic (Galey *et al*., 1996) [23], such that we considered this case to be closed.

### 3.3. *When microscopy remains vague*: Goat ruminal fillings

**Fig 4:**
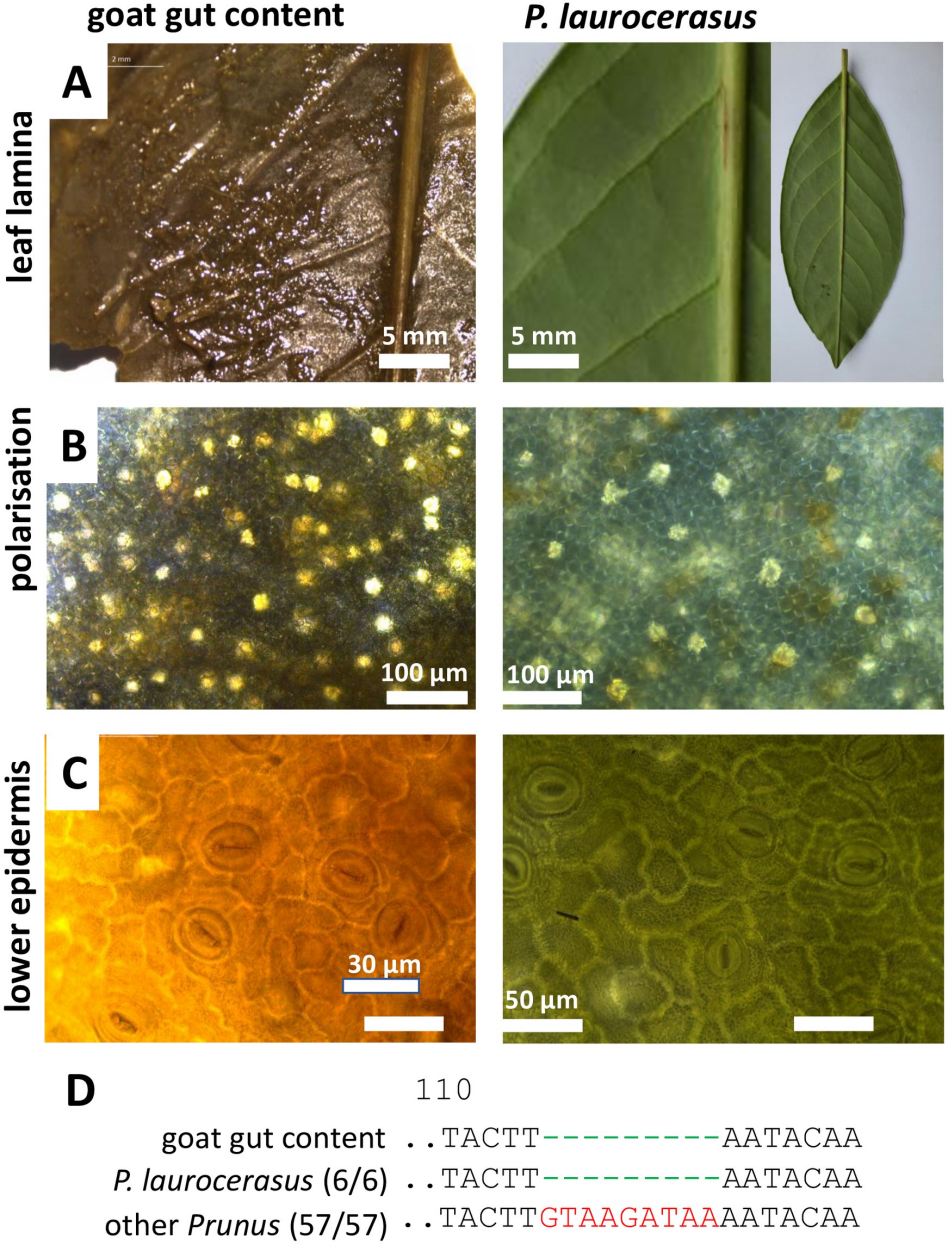
Morphological and anatomical comparison between macerated leaf fragments from the stomach of a goat (case 2) and a reference plant for *Prunus laurocerasus* from the KIT Botanical Garden. **A** lamina with characteristic parallelilty of lateral veins **B** adaxial view with crystal druses and square-shaped mesophyll cells visualised by polarisation microscopy. **C** anemocytic stomata the lower epidermis. **D** diagnostic fingerprints in the *trnH-psbA igs* marker.

The second case was more difficult to solve. A goat, which at the time of its discovery was already partially decomposed and showed symptoms of a catarrhal enteritis produced ruminal fillings that were highly homogenised, such that only few fragments of masticated leaves could be discovered. From their venation, they were possible originating from cherry laurel (*Prunus laurocerasus*), but this venation pattern (Fig 4A) is quite widespread and far less specific than the parallel venation of *N*. *oleander* in the previous case. Also, microscopic diagnostics was not very helpful. While the square-like pavement cells of the adaxial epidermis and the crystals visible upon polarisation microscopy resembled the features seen in the leaf of the reference plant (Fig 4B), both traits are found in many other plants as well. The same holds true for the anemocytic stomata that could be seen on the abaxial surface of the leaf fragments (Fig 4C). Thus, the conclusion derived from anatomical inspection that this goat had eaten leaves of cherry laurel was built on shaky ground. Fortunately, it was possible to generate a *trnH-psbA igs* barcode of sufficient quality (S4 Data), and the phylogenetic tree inferred from the sequences placed the sequence recovered from the ruminal filling close to that of the reference plant for *P*. *laurocerasus* as well as to the sequences found in public databases (S3 Fig). The sequences were also well delineated from all sequences available for other members of the genus *Prunus*. Moreover, the sequence drawn from the ruminal filling showed a specific deletion of 10 bp at position 114 of the alignment that was found in all sequences from *P*. *laurocerasus* (including the authenticated reference plant) but was absent from all 57 sequences from *Prunus* species different from *P*. *laurocerasus*. Thus, this DNA fingerprint lent very strong support to our hypothesis that the goat had ingested leaves from *P*. *laurocerasus*, which contains amygdalin and its degradation product prunasin, which in the acidic environment of the ruminal lumen will be converted to benzaldehyde and the highly toxic hydrogen cyanide (for a recent review see Jaszczak-Wilke et al., 2021) [24].

**Fig 5:**
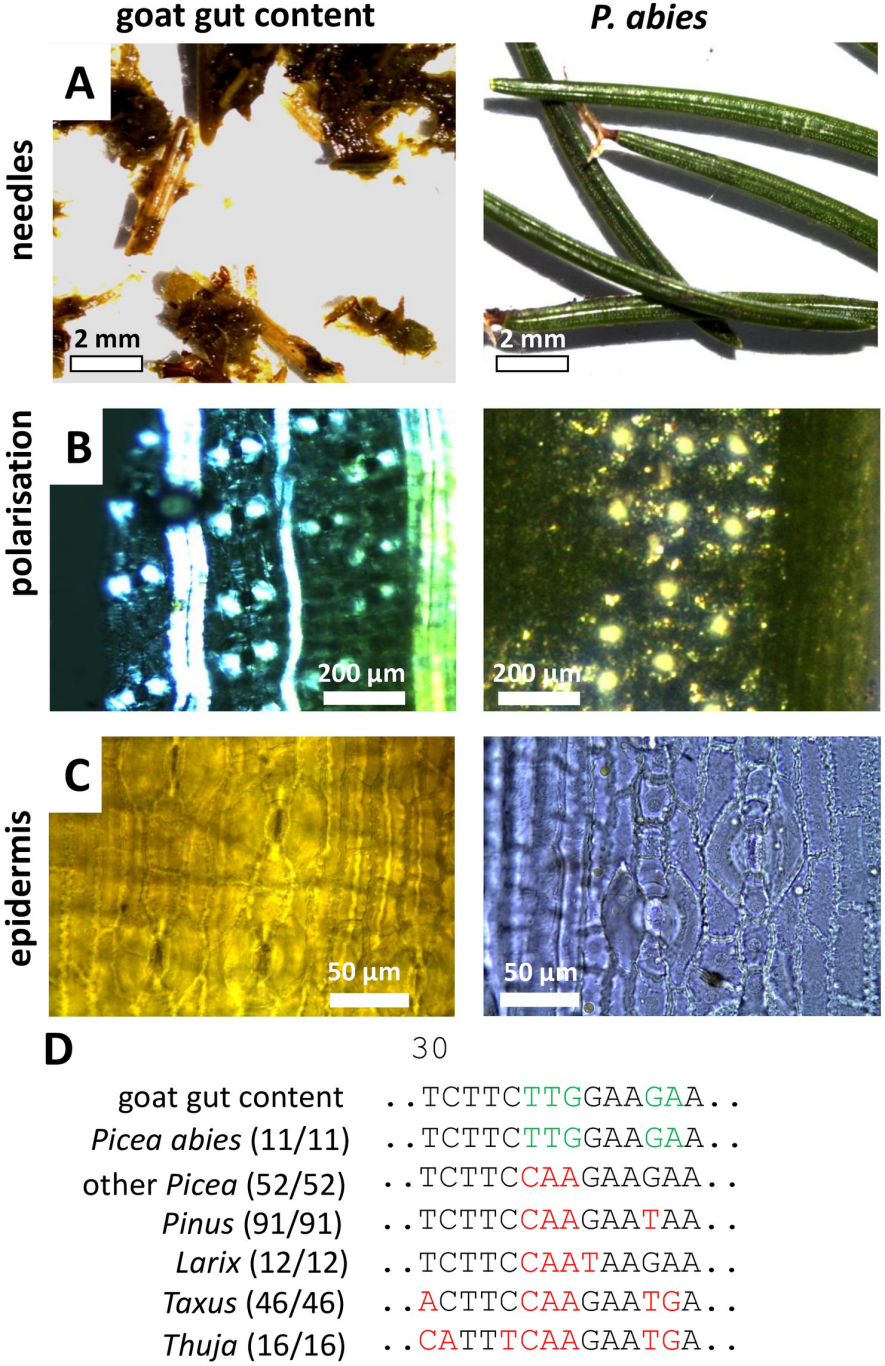
Morphological and anatomical comparison between macerated needle fragments from the stomach of a goat (case 3) and a reference plant for *Picea abies* from the KIT Botanical Garden. **A** macroscopical aspect of needles **B** birefringency of stomata visualised by polarisation microscopy. **C** bright-field image of epidermis with paracytic stomata. **D** diagnostic fingerprints in the *trnH-psbA igs* marker.

The third case, also a deceased goat, added a further level of challenge. Here, the ruminal fillings were homogenised to such a degree that only a few needle-like fragments could be recovered (Fig 5A). The polarisation microscopy (Fig 5B) revealed the presence of birefringent crystals, but similar crystals are found in numerous plants and are not suited as *a-priori* anatomical trait. The rows of cells with wavy cell walls and the paracytic stomata (Fig 5C) are also a trait shared with many other taxa. We, therefore, amplified the *trnH-psbA igs* marker, which matched the sample from the goat gut with the common spruce, *Picea abies* (S4 Fig), delineating it clearly from other conifers that occur in that part of Germany. The results from the phylogeny were supported by two very specific fingerprints at position 35 of the alignment (S5 Data), which was shared by the goat sample with all 10 sequences recovered for *P*. *abies* from public databases as well as an authenticated reference plant (Fig 5D). This fingerprint was absent from all 52 homologues of other *Picea* species found in GenBank, as well as of the genera *Pinus* (92 sequences), *Larix* (12 sequences), *Taxus* (46 sequences), and *Thuja* (16 sequences). When we verified the anatomical features of a reference plant for *P*. *abies* (Fig 5A–5C), we found a complete congruence with the traits seen in the remnants recovered from the goat gut, which was consistent with our conclusion that this animal had eaten spruce needles. However, this anatomical match was validated only *a posteriori* to the genetic assay and would not have been sufficient to reach this diagnosis if taken alone, because these morphological traits are not very specific and shared with numerous plants, even from completely unrelated taxa.

### 3.4. *When morphology becomes ambiguous*: The case of a *Pieris* hybrid

**Fig 6:**
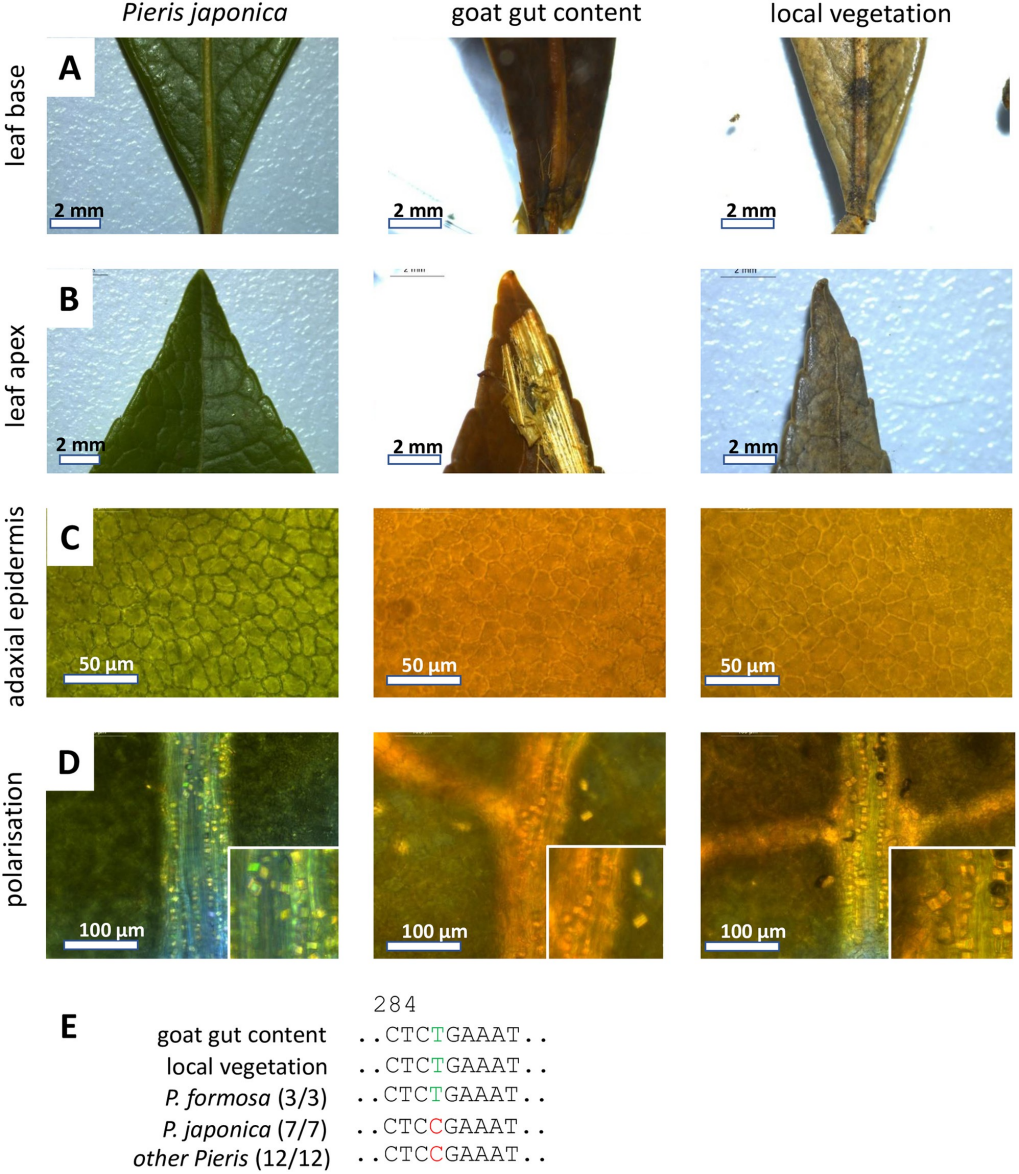
Morphological and anatomical comparison between macerated leaf fragments from the stomach of goat (case 4), samples of local vegetation, and a reference plant for *Pieris japonica* from the KIT Botanical Garden. **A** Leaf base. **B** Leaf tip **C** Adaxial epidermis with polygonal pavement cells. **D** Crystals at leaf veins visualised by polarisation microscopy. **E** diagnostic fingerprints in the trnH-psbA marker.

Sometimes, although morphological authentication is possible, the result remains somewhat ambiguous. This was the case for a third deceased goat, where gut content and local vegetation collected at the site of the incident, both by morphology and anatomical details (Fig 6A–6D) led immediately to the conclusion that this animal had eaten leaves of Japanese andromeda (*Pieris japonica*). However, the comparison with an authenticated reference from the KIT Botanical Garden, revealed that, while the anatomical details were matched, the morphology of the leaf was similar, but not identical. Especially leaf base and leaf apex of the specimen recovered from the goat was more pointed as to be expected for *Pieris japonica*, pointing to a different species of this genus. A molecular phylogeny based on the trnH-psbA marker (S5 Fig) placed the sequence recovered from the animal gut and the local vegetation outside of *P*. *japonica*, basal to the clade formed by *P*. *formosa*, but also nearby several alternative members of the genus. Likewise, the morphological details given by the Flora of China, such as the lack of indentation in the lower leaf margin and the shorter petioles, were deviating from that to be expected for *P*. *formosa* and rather spoke in favour of *P*. *japonica*. The e-values from a BLAST search were all fairly similar and did not help to assign these sequences to a particular species within the genus. We wondered whether specific fingerprints might help to decide the case. In fact, we were able to identify a Single Nucleotide Polymorphism at position 287 (S6 Data) in the alignment (Fig 6E) that was unique for *P*. *formosa* and absent from *P*. *japonica* as well as from all sequences available for other *Pieris* species. This fingerprint was found in both, the sequences recovered from the goat gut as well as from the local vegetation. Since the trnH-psbA marker is inherited maternally, we arrived at the conclusion that the goat had eaten material from a hybrid plant deriving from a *P*. *japonica* father and a *P*. *formosa* mother. In fact, such a hybrid is commonly sold in German garden centres, and, thus, the goat, with high probability, had been victim of ornamental plants that had gone feral.

### 3.5. *When morphology eclipses*: Diagnosing cattle and horse poisonings

**Fig 7:**
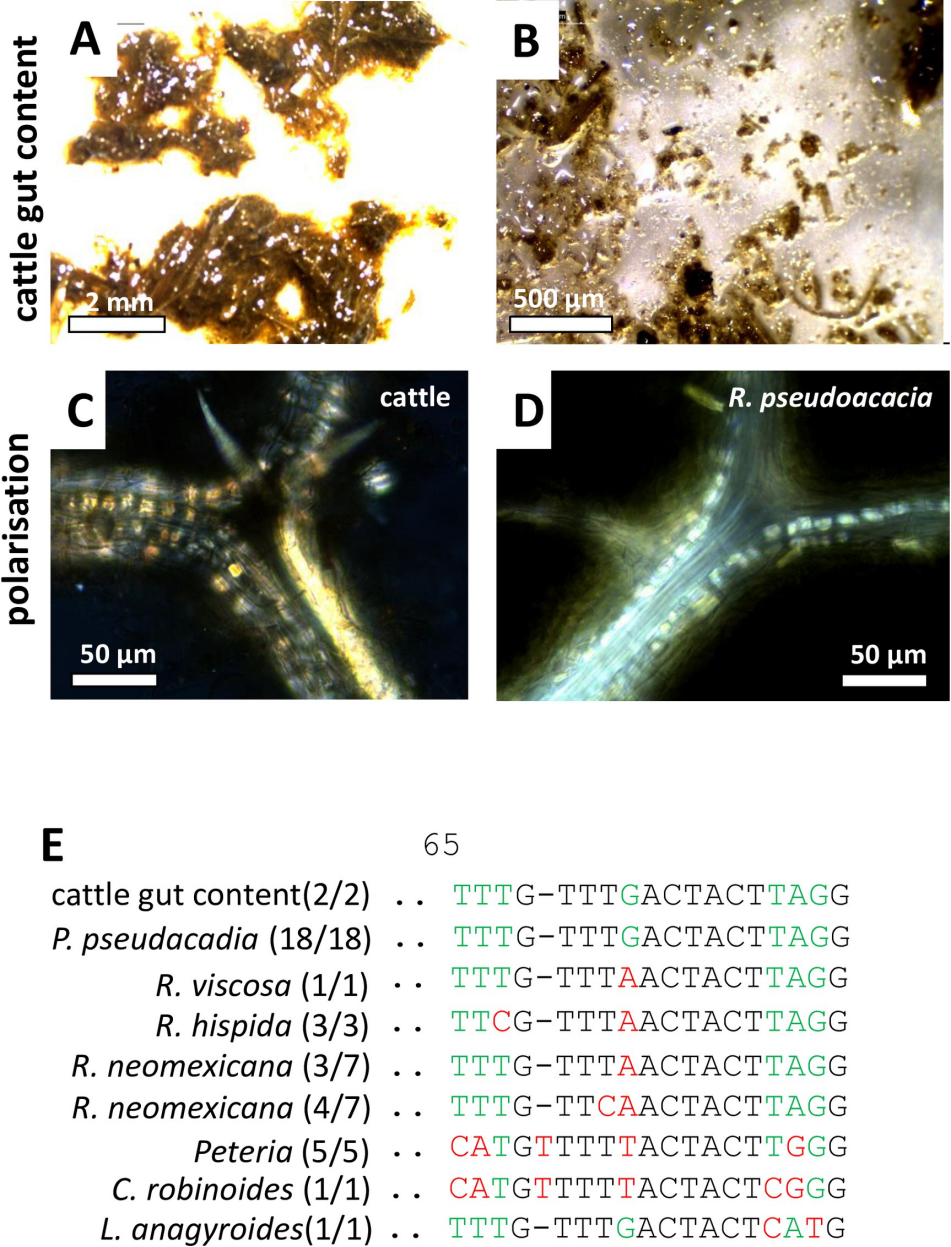
Morphological and anatomical comparison between macerated leaf fragments from the stomach of a cattle (case 4) and a reference plant for *Robinia pseudoacacia*. **A, B** macroscopical aspect of the cattle samples **C, D** birefringency of crystals along leaf veins visualised by polarisation microscopy. **E** diagnostic fingerprints in the *ITS* marker.

Diagnosis of cattle or horse poisonings are far from trivial, because, here, the anatomical features of the plants are homogenised to a degree, where no microscopic diagnosis is possible. Here, genetic barcodes have probably the strongest potential as illustrated by the following two cases:

A deceased cattle suffering a complete breakdown of the inner organs including perirenal edema, hemorrhage, diffuse renal tubular degeneration, parenchymal congestion, and lung edema (Table 4) provided only completely homogenised ruminal fillings (Fig 7A and 7B) that contained only tiny and scarce fragments of leaf material that under polarised light showed cubic, birefringent crystals along the veins (Fig 7C) that did not suffice for any diagnostic assignment, since such crystals occur in many plants as seen already in the current study. Amplificates obtained for the *ITS* marker placed the sample into the species *Robinia pseudoacacia* (black locust) delineating from other species of the same genus, albeit at low insufficient resolution (S6 Fig). However, a specific fingerprint at position 65 of the alignment (S7 Data) was found only in the cattle and in all available sequences for *R*. *pseudacacia* (Fig 7E), while even the closest relatives within the genus *Robinia* differed in at least one nucleotide. Since the cattle seemed to have ingested Black Locust, we wondered, whether the typical birefringent crystals along the veins were present in reference material from this species. This implication could be verified (Fig 7D), however, only *post hoc* (it would not have been possible to diagnose Black Locust just on the base of the crystals without the genetic data).

**Fig 8:**
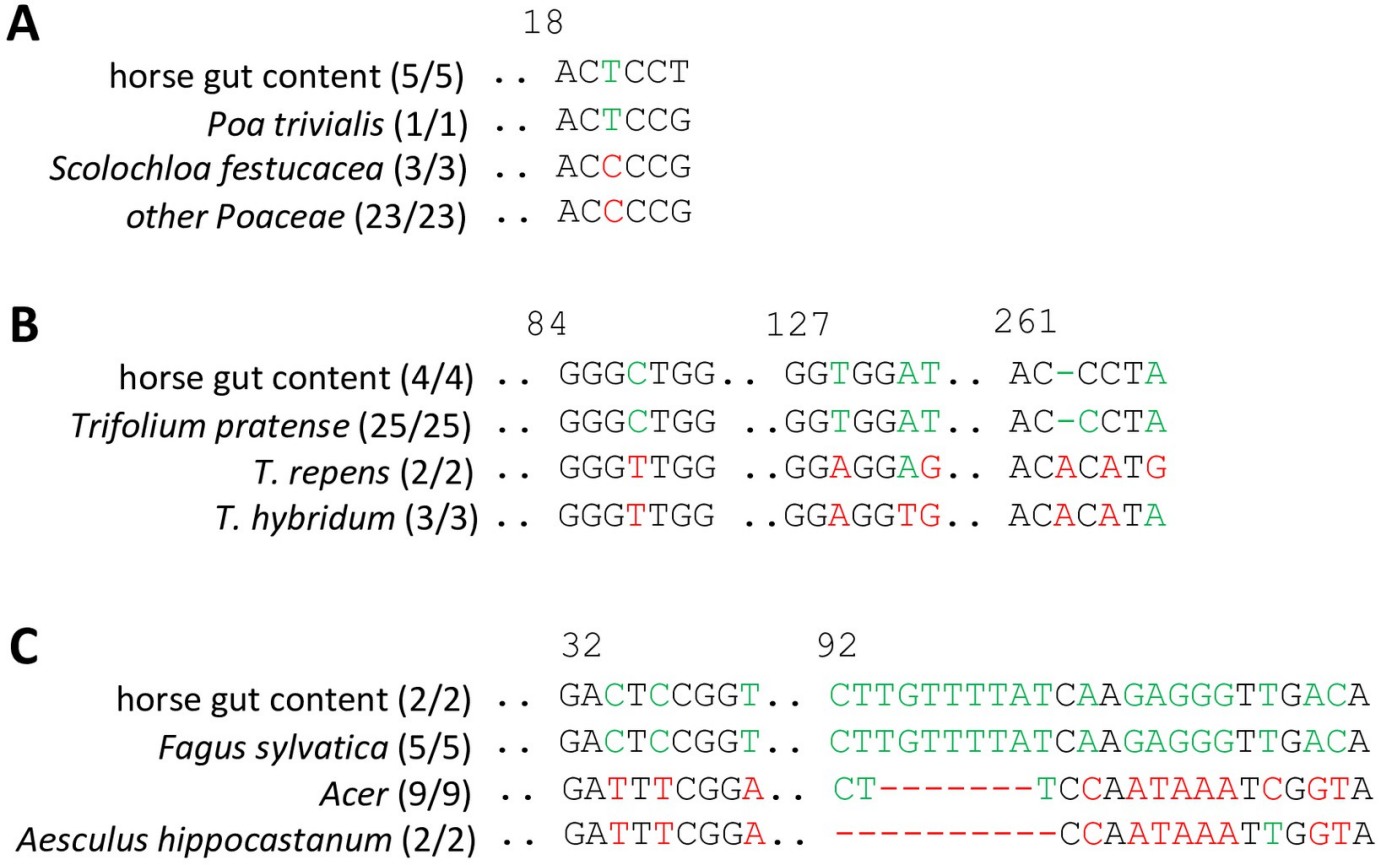
DNA barcoding fingerprints recovered for the gut content of a horse. **A** Diagnostic fingerprint in the *rbcL* marker of the horse samples indicative of *Poa trivialis*, *Scolochloa festucacea* (listed as best hit in the NCBI database) and other Poaceae that are common on meadows and pastures in Germany. **B** Diagnostic fingerprints in the *ITS1* marker of the horse samples indicative of *Trifolium pratense* versus *T*. *repens* and *T*. *hybridum* (as alternative common clover species on meadows and pastures in Germany). C Diagnostic fingerprints in the psbA-trnH marker of the horse samples indicative of *Fagus sylvatica* versus the three species of *Acer* occurring in Germany, and *Aesculus hippocastanum*.

Along the gradient demonstrated in the current study, the case of a poisoned horse represents the ultimate stage of homogenisation. Here, the intestinal content was void even of remnants that could be used for microscopical diagnostics, such that genetic barcoding remained as single approach. Since it was clear that this will be a challenging sample, we started off with the plastidic rbcL marker, which is robust and often works even in cases, where other markers fail to become amplified. In fact, we were able to recover a fragment, which was of good sequence quality and clustered into the Poaceae, but outside of known genera (S7 Fig). This fragment was located in a relatively conserved part of the marker, which might explain the poor resolution (S8 Data). A BLAST search in GenBank delivered *Scolochloa festucacea* as best hit, but only with a moderate score of ~95%. This grass has been reported to contain indole alkaloids with anti-acetylcholinic activity and, thus, might be toxic, if ingested in larger quantities. However, despite a long bibliographic search, no reports on animal intoxication could be detected. A closer look into the alignment revealed a specific SNP at position 20 of the alignment, where the sequences recovered from the horse guts differed from *S*. *festucacea*, as well as of all other available Poaceae sequences (Fig 8A). This fingerprint was shared with the sequence from *Poa trivialis*, which was also matching in the other regions of the sequence. While this congruence lends strong support to the hypothesis that this horse had ingested *P*. *trivialis*. However, *P*. *trivialis* is a common pasture species and by no means qualifies as cause for the poisoning.

We tested then the nuclear *ITS* marker on the horse samples and the phylogenetic tree inferred from the sequence alignment (S9 Data) placed the sequences recovered from the horse guts clearly into the species *Trifolium pratense*, white clover (S8 Fig). Again, three specific fingerprints allowed a clear delineation of these sequences from the related species *Trifolium repens* and *Trifolium hybridum* that, similar to white clover, are common pasture plants (Fig 8B). Again, while it is quite plausible that the horse had eaten white clover, it is highly unlikely that the intoxication originated from that plant.

In a third attempt, we isolated the *trnH-psbA* marker. The resulting alignment (S10 Data) with the best hits recovered from a BLAST search should a complete match with the sequence homologues from beech (*Fagus sylvatica*) and this impression was confirmed by the phylogenetic tree inferred thereof (S9 Fig). Two very specific fingerprints showed perfect congruence with *F*. *sylvatica*, while related tree species that occur commonly in Germany and are known to cause horse poisonings, such as *Acer* species or horse chestnut, differed significantly, for instance by gaps in the alignment (Fig 8C).

## 4. Discussion

In the current work, we have explored the potential of genetic barcodes to solve cases of lethal animal poisoning, moving along a gradient, where anatomical traits were progressively missing, due to digestive activity. While there exist already catalogues listing genetical information to detect poisonous plants (Wang *et al*., 2021 [12]; Nithaniyal *et al*., 2021) [13], the evidence that such information can be successfully employed in real-world applications is missing so far. We have, therefore, based this study exclusively on real-world cases from the everyday routine of diagnostic veterinary pathology of the State of Baden-Württemberg, Southwest Germany. To the best of our knowledge, this represents the first application of genetic barcoding on veterinary forensics. Furthermore, while taxon assignment is conventionally derived from statistical e-values deriving from the BLAST search (often leading to ambiguities, when the e-values are not very high and comparable between different candidate taxa), we use here taxon-specific single-nucleotide polymorphisms as diagnostic fingerprints that help to reach a clear hypothesis even in cases, where e-values leave the decision ambiguous. Due to the applied nature of this study, the discussion will focus on methodological aspects. What is the best strategy to infer from a recovered sequence a hypothesis on the intoxication cause? What are the limitations of this approach and how can we breach them in future work? What is the potential of the approach and what type of research does it enable that had not been accessible so far?

### 4.1. From the corpus delicti to the hypothesis on the intoxication cause

Following a gradient of progressive dissolution of anatomical markers, we were able to come up with plausible and precise hypotheses on all cases of intoxication of this study. However, this was not always successful at the first attempt.

While, in the first case of the study, the combination of morphology and specific SNPs in the *trnH-psbA* marker provided unequivocal proof that the deceased alpaca had ingested leaves of *Camellia japonica* (Fig 2), this plant did not qualify as plausible cause of death. Although *Camellia* species are known as rich source for secondary compounds (for review see Texeira and Sousa, 2021) [25], neither for Japanese camelia nor its Chinese sister, *C*. *sinensis*, the tea plant, any toxicity has been reported so far. Thus, while it is clear that the leaf material in the gut of the deceased animal was from *C*. *japonica*, it is not likely that the alpaca died from Japanese Camelia. Only our second attempt, focussing on a few leaf fragments from a different plant, led to the more plausible explanation that the animal had deceased from *Nerium oleander* (Fig 3). Again, both the anatomical and the genetic evidence was congruent, not leaving space for any ambiguity. The reported symptoms were matching this hypothesis. Acute death linked with pulmonary edemas are characteristic symptoms of oleander poisoning. This is linked with cardenolide-type cardiac glycosides, such as oleandrine. Already 20 ng/ml are sufficient to cause acute death of humans (Wasfi *et al*., 2008) [26]. The mode of action is linked to inhibition of the sodium-potassium antiporters in the cardiac myocytes (Langford and Boor, 1996) [4], which through interfering with the sodium-calcium antiporter leads to accumulation of cytosolic calcium, which cannot any longer be extruded, culminating in heart failure. In case of *Pieris* (Fig 6), the presence of diterpenes, known as grayanotoxins, sometimes also andromedotoxins, implicated in lethal cases of goat poisoning reported in the literature (Puschner *et al*., 2001) [27] represent an important element to the forensic hypothesis that the goat indeed died from intoxication with this species. Similarly, the well-known toxicity of toxalbumins such as robin and phasin, eliciting a deregulated immune response with agglutinated erythrocytes followed by hemorrhagic collapse (Uhlig *et al*., 2007) [28] strongly corrobates the forensic hypothesis that the cattle had died from ingesting black locust (Fig 7).

What this case is teaching us: a *corpus delicti*, as convincing it may appear, does not lead *per se* to a viable hypothesis, one always needs to integrate additional information beyond the authentication, to avoid being misled.

This conclusion is even more accentuated, when the evidence from the sample is one-dimensional, for instance, because no anatomical information can be recovered due to advanced homogenisation of the gut content. This is well illustrated by the last case in our series, the deceased horse (Fig 8). Here, three attempts were needed to arrive at an explanation, because the first two amplifications, using the *rbcL* and the *ITS1* markers, led to commonplace food plants (*Poa trivialis*, *Trifolium pratense*) that are clearly void of any toxicity. Instead, the third attempt, using *trnH-psbA igs* as marker, led to a reasonable hit. Beechnuts contain trimethylamine (fagin), especially those coming from the European beech, and they are known to be poisonous to horses (Wilkens and Cranwell, 1990) [29]. Thus, our working hypothesis for this case was that the horse had grazed *Poa trivialis* and *Trifolium album* which belong to its common diet. However, apparently, it had also taken up some beechnuts that may have fallen to the ground and probably are responsible for the lethal intoxication, the lethal dose (0.3–1 kg) being sufficiently low to realistically qualify as the cause of death.

Overall, one can conclude that anatomical and barcoding markers were successful in identifying the ingested plant. However, this is just a *corpus delicti*, not more. To arrive at a plausible forensic hypothesis, this *corpus delicti* is not sufficient. It needs to be combined with additional knowledge, for instance on the toxicity of the respective plant, on significant pathomorphological (necropsy) findings or the reported symptoms of the clinical case/course.

### 4.2. Sequence-based fingerprinting beats mere statistics

The usual approach to identify a source species from sequence information is to do a BLAST search in public databases listing the best hits based on their Expect value, commonly known as e-value. This parameter describes the number of hits one would expect for random sequences giving the match with the query sequence (National Centre for Biotechnology Information, 2022) [30]. This value depends on the number of available entries, but also on the length of the sequence. It is a statistical value which is of generic nature–a long barcoding region that is conserved may mask the few informative sites leading to a situation, where several hits are listed with identical or very similar e-values. Especially in plants, where the taxon gap is much lower than in animals, while intra-taxon variability is higher (Fazekas *et al*., 2009) [9], the BLAST search often leads to ambiguous results that are not really helpful. We have adopted, therefore, a different approach, focussing on informative motives in the alignment, rather than on overall genetic distance of the barcode. It is possible to discriminate even neighbouring taxa by Single Nucleotide Polymorphisms (SNPs) at specific sites, if one can show that this SNP is supported by a sufficient number of accessions from the respective pair of taxa. Basically, the resolution power of this approach excels that of the e-value because it considers the position in the alignment and, in many cases, details of the adjacent positions constituting a specific fingerprint for the respective taxon. These details constitute a specific quality *in sensu* Remane (1971) [31] that is far more informative than a number based on quantitative statistics. The criterion of specific quality has not only been useful in establishing morphological homology, but is also a central tool to decide whether an observed sequence difference derives from molecular convergence or from a true homology (Nick, 2018) [32]. In fact, using this criterion of specific quality it is possible to separate informative sequence signatures from intra-taxon variation and arrive at a clear conclusion even in cases, where the mere e-value gives ambiguous results. For instance, this strategy has been successfully employed to resolve cases, where, due to the ambiguity of vernacular nomenclature, different taxa are traded under the same name, such as in case of the Holy Basil *Tulsi* (Jürges *et al*., 2018) [33] or the Peruvian Amaranth *kiwicha* (Kanbar *et al*., 2022) [34].

### 4.3 Limitations and potentials

Although the use of sequence-based fingerprints was able to infer a feasible forensic hypothesis for all the cases addressed in the current study, the limitations of this approach became also clearly manifest. There are basically three drawbacks to consider for the further development of this strategy:

#### The impact of potential sampling bias

Both, the analysis of anatomical details in plant remnants, as well as the amplification of barcodes depends on the location within the gut system. This can lead to misinterpretations, if a small quantity of toxic plant material had been ingested along with quantitatively dominating feed plants. In our study, this situation is illustrated by the case of the poisoned horse (Fig 8), where the first two attempts to amplify a genetic barcode led to two commonplace pasture species (*Poa trivialis*, *Trifolium pratense*) that are expected in the gut content of a free-grazing horse. Also, the first case with the deceased alpaca, the first hit, *Camellia japonica* (Fig 2), was not leading to a plausible explanation, and only the recovery of the scarce remnants of a second plant, *Nerium oleander* (Fig 3) produced a viable forensic hypothesis. Thus, in the current study, sampling bias of toxic, but not abundant plant remnants led to the need to repeat the approach several times till it led to success (using forensic plausibility as criterion). To address samplings bias in a more systematic manner, one might explore strategies probing at a given number of sites in the gut, or by mixing a larger number of samples in a standardised manner to reach symmetric representation (Elias *et al*., 2012) [35].

#### Handling mixtures

In those cases, where particular remnants could be recovered, it was possible to amplify and sequence the barcode by Sanger sequencing. With progressive homogenisation of the gut content this is getting more difficult because mixtures of templates will lead to blurred sequences that cannot be unequivocally assigned to a species. This limitation could be breached by analysing the amplicons by Next-Generation Sequencing, an approached widely used in molecular ecology, also on gut contents to characterise the food spectrum of an animal (for review see Pompanon *et al*., 2012) [36], which at the same time would also allow to assess the frequency distribution of individual species in the gut content. The resolving power of this strategy could be improved if the BLAST search routine of the reconstructed sequence information would be combined with sequence-based fingerprinting as discussed above. Symmetric sampling (see above) would be a necessary precondition, however.

#### Do not detect only, quantify

Similar to the vast majority of studies on animal poisoning, the route of the current investigation was of qualitative nature. We were able to identify the poisonous plant, which in many cases is sufficient to come up with a forensic hypothesis. However, if we take Paracelsus seriously (*dosis sola venenum facit*), we cannot stop at a detection method that is merely quantitative. We should, therefore, develop the current method towards quantification. This is possible using real-time qPCR, because the amount of template DNA for a given species can be determined from the C_t_ value, an approach that has been successfully employed to check declared berry fruit content in commercial fruit preparations (An *et al*., 2019) [37], and, thus, should also be amenable to gut contents.

#### Towards an epidemiology of animal poisonings

Forensics is an anecdotal approach, each case is specific and to come up with a hypothesis, one needs to rely upon incomplete and mostly circumstantial evidence. To detect general patterns is very difficult, because most cases of plant-based animal poisoning remain unresolved. The use of genetic markers that has been elaborated in the current study significantly increases the frequency of forensic hypotheses, especially in animals where the digestive system leads to rapid homogenisation of the ingested material. This increase in the success rate allows the transition from individual cases to the statistical level. In other words, genetic diagnostics paves the way for epidemiological studies. For instance, it is completely unclear, how widespread beech poisoning of horses actually is, because the majority of cases probably goes unexplained. Now, it has become possible to do a comparative study and collect samples from horse poisonings all over the country and find out, what proportion of those contains *Fagus sylvatica*. It might well be that such epidemiological studies uncover patterns that have remained obscure due to the circumstantial nature of the cases and the incomplete clarification of their cause.

## Supporting information

S1 Fig Molecular phylogeny inferred for the *trnH-psbA* marker from 102 sequences covering the genus *Camellia* along with the sequence recovered from the gut content of the deceased alpaca using the Neighbour-Joining algorithm. (PPTX)

S2 Fig Molecular phylogeny inferred for the *trnH-psbA* (A) and the *ycf1b* (B) marker from 16 sequences covering the Apocynaceae family along with the sequence recovered from the gut content of the deceased alpaca using the Neighbour-Joining algorithm. (PPTX)

S3 Fig Molecular phylogeny inferred for the *trnH-psbA* marker from 65 sequences covering the genus *Prunus* family along with the sequence recovered from the gut content of the deceased goat using the Neighbour-Joining algorithm. (PPTX)

S4 Fig Molecular phylogeny inferred for the *trnH-psbA* marker from 28 sequences covering different conifer families along with the sequence recovered from the gut content of the deceased goat using the Neighbour-Joining algorithm. (PPTX)

S5 Fig Molecular phylogeny inferred for the *trnH-psbA igs* marker from 28 sequences covering the genus *Pieris* and neighbouring genera of the Ericaceae along with the sequence recovered from the gut content of the deceased goat and the local vegetation using the Neighbour-Joining algorithm. (PPTX)

S6 Fig Molecular phylogeny inferred for the *ITS* marker from 34 sequences covering the genus *Robinia* and neighbouring genera of the Fabaceae along with two sequences recovered from the gut content of the deceased cattle using the Neighbour-Joining algorithm. (PPTX)

S7 Fig Molecular phylogeny inferred for the *rbcL* marker from 28 sequences covering the Poaceae that are common on meadows and pastures in Germany along with five sequences recovered from the gut content of the deceased horse using the Neighbour-Joining algorithm. (PPTX)

S8 Fig Molecular phylogeny inferred for the *ITS1* marker from 31 sequences covering clovers that are common on meadows and pastures in Germany along with four sequences recovered from the gut content of the deceased horse using the Neighbour-Joining algorithm. (PPTX)

S9 Fig Molecular phylogeny inferred for the *trnH-psbA igs* marker from 16 sequences covering beeches and related trees that are common on meadows and pastures in Germany along with two sequences recovered from the gut content of the deceased horse using the Neighbour-Joining algorithm. (PPTX)

S1 Data Alignment in FASTA format for the *trnH-psbA igs* marker from 102 sequences covering the genus *Camellia* along with the sequence recovered from the gut content of the deceased alpaca. (TXT)

S2 Data Alignment in FASTA format for the *trnH-psbA igs* from 16 sequences covering the Apocynaceae family along with the sequence recovered from the gut content of the deceased alpaca. (FAS)

S3 Data Alignment in FASTA format for the *ycf1b* from 16 sequences covering the Apocynaceae family along with the sequence recovered from the gut content of the deceased alpaca. (FAS)

S4 Data Alignment in FASTA format for the *trnH-psbA igs* marker from 65 sequences covering the genus *Prunus* family along with the sequence recovered from the gut content of the deceased goat. (FAS)

S5 Data Alignment in FASTA format for the *trnH-psbA igs* marker from 28 sequences covering different conifer families along with the sequence recovered from the gut content of the deceased goat. (FAS)

S6 Data Alignment in FASTA format for the *trnH-psbA igs* marker from 28 sequences covering the genus *Pieris* and neighbouring genera of the Ericaceae along with the sequence recovered from the gut content of the deceased goat and the local vegetation. (FAS)

S7 Data Alignment in FASTA format for the *ITS* marker from 34 sequences covering the genus Robinia and neighbouring genera of the Fabaceae along with two sequences recovered from the gut content of the deceased cattle. (FAS)

S8 Data Alignment in FASTA format for the *rbcL* marker from 28 sequences covering the Poaceae that are common on meadows and pastures in Germany along with five sequences recovered from the gut content of the deceased horse. (FAS)

S9 Data Alignment in FASTA format for the *ITS1* marker from 31 sequences covering clovers that are common on meadows and pastures in Germany along with four sequences recovered from the gut content of the deceased horse. (FAS)

S10 Data Alignment in FASTA format for the *trnH-psbA igs* marker from 16 sequences covering beeches and related trees that are common on meadows and pastures in Germany along with two sequences recovered from the gut content of the deceased horse. (FAS)

## Abbreviations

rbcL: Ribulose-1,5-Bisphosphate Carboxylase Large Subunit
trnH-psbA: histidine transfer RNA (trnH) photosystem II protein D1 precursor (psbA) intergenic spacer

## References

[1] McFarland SE, Mischke RH, Hopster-Iversen C, von Krueger X, Ammer H, Potschka H, et al. Systematic account of animal poisonings in Germany, 2012–2015. Vet Rec. 2017; 180: 327. doi: 10.1136/vr.103973 28235786

[2] Berny P, Caloni F, Croubels S, Sachana M, Vandenbroucke V, Davanzu F, et al. Animal poisoning in Europe. Part 2: companion animals. Vet J. 2019; 183: 255–259. 10.1016/j.tvjl.2009.03.034 19553146

[3] Guitart R, Croubel S, Caloni F, Sachana M, Davanzo F, Vandenbroucke V, et al. Animal poisoning in Europe. Part 1: farm livestock and poultry. Vet J. 2010; 183: 249–254. doi: 10.1016/j.tvjl.2009.03.002 19359202

[4] Langford S, Boor P. Oleander toxicity: an examination of human and animal toxic exposures. Toxicol. 1996; 109: 1–13. doi: 10.1016/0300-483x(95)03296-r 8619248

[5] Moyano MR, García A, Rueda A, Molina AM, Méndez A, Infante F. *Echium vulgare* and *Senecio vulgaris* poisoning in fighting bulls. J Vet Med A. 2006; 53: 24–25. 10.1111/j.1439-0442.2006.00780.x 16411904

[6] Bofill FX, Bofill J, Such G, Piqué E, Guitart R. Intoxicación por contaminación del maíz con *Datura stramonium*: ¿Un problema creciente para el ganado? Revista Toxicol. 2007; 24: 56–58.

[7] Antoniou V, Zantopoulos N, Samouris G, Ioannidou M. Impacts of toxic substances in sheep and goats in northern Greece. Animal Sci Rev. 2019; 34: 21–28.

[8] CBOL Plant Working Group. A DNA barcode for land plants. Proc Natl Acad Sci USA. 2009; 106: 12794–12797. doi: 10.1073/pnas.0905845106 19666622 PMC2722355

[9] Fazekas AJ, Kesanakurti PR, Burgess KS, Percy DM, Graham SW, Barrett SCH, et al. Are plant species inherently harder to discriminate than animal species using DNA barcoding markers? Mol Ecol Res. 2009; 9 (Suppl 1): 130–139. doi: 10.1111/j.1755-0998.2009.02652.x 21564972

[10] Galimberti A, De Mattia F, Losa A, Bruni I, Federici S, Casiraghi M, et al. DNA barcoding as a new tool for food traceability. Food Res Int. 2013; 50: 55–63.

[11] Ichim MC. The DNA-based authentication of commercial herbal products reveals their globally widespread adulteration. Front Pharmacol. 2019; 10: 1227. doi: 10.3389/fphar.2019.01227 31708772 PMC6822544

[12] Nithaniyal S, Majumder S, Umapathy S, Parani M. Forensic application of DNA barcoding in the identification of commonly occurring poisonous plants. J Forensic Leg Med. 2021; 78: 102126. doi: 10.1016/j.jflm.2021.102126 33556892

[13] Wang J, Zhao J, Yu W, Wang S, Bu S, Shi X et al. Rapid Identification of Common Poisonous Plants in China Using DNA Barcodes. Front Ecol Evol. 2021; 9: 698418.

[14] Valentini A, Pompanon F, Taberlet P. DNA barcoding for ecologists. Trends Ecol Evol. 2009; 24: 110–117. doi: 10.1016/j.tree.2008.09.011 19100655

[15] Kartzinel TR, Chen PA, Coverdale TC, Erickson DL, Kress WJ, Kuzmina ML, et al. DNA metabarcoding illuminates dietary niche partitioning by African large herbivores. Proc Natl Acad Sci USA. 2015; 112: 8019–8024. doi: 10.1073/pnas.1503283112 26034267 PMC4491742

[16] Flora of China. http://www.efloras.org/flora_page.aspx?flora_id=2, accessed 11.04.2022.

[17] Aichele D, Schwegler H-W. Die Blütenpflanzen Mitteleuropas. 1. ed. Stuttgart: Franckh- Kosmos; 1994.

[18] Sang T, Crawford DJ, Stuessy TF. Chloroplast DNA phylogeny, reticulate evolution, and biogeography of *Paeonia* (Paeoniaceae). Am J Bot. 1997; 84: 1120–1136. 21708667

[19] Tate JA, Simpson BB. Paraphyly of tarasa (Malvaceae) and diverse origins of the polyploid species. System Bot. 2003; 28: 723–737.

[20] Dong WP, Xu C, Li CH, Sun JH, Zuo YJ, Shi S, et al. *ycf1*, the most promising plastid DNA barcode of land plants. Sci Rep. 2015; 5: 8348. 25672218 10.1038/srep08348 PMC4325322

[21] Chiou SJ, Yen JH, Fang CL, Chen HL, Lin TY. Authentication of medicinal herbs using PCR-amplified ITS2 with specific primers. Planta Medica. 2007; 73: 1421–1426. doi: 10.1055/s-2007-990227 17909989

[22] Kress WJ, Erickson DL. A two-locus global DNA barcode for land plants: the coding *rbcL* gene complements the non-coding *trnH-psbA* spacer region. PLoS ONE. 2007; 2: e508. 17551588 10.1371/journal.pone.0000508 PMC1876818

[23] Galey FD, Holstege DM, Plumlee KH, Tor E, Johnson B, Anderson ML, et al. Diagnosis of oleander poisoning in livestock. J Vet Diagn Invest. 1996; 8: 358–364. doi: 10.1177/104063879600800314 8844581

[24] Jaszczak-Wilke E, Polkowska Ż, Koprowski M, Owsianik K, Mitchell AE, Bałczewski P. Amygdalin: Toxicity, Anticancer Activity and Analytical Procedures for Its Determination in Plant Seeds. Molecules. 2021; 13: 2253. doi: 10.3390/molecules26082253 33924691 PMC8069783

[25] Teixeira AM, Sousa C. A Review on the Biological Activity of Camellia Species. Molecules. 2021; 26: 2178. doi: 10.3390/molecules26082178 33918918 PMC8069326

[26] Wasfi I, Zorob O, Alkatheeri N, Alawadhi A. A fatal case of oleandrin poisoning. Forens Sci Int. 2008; 179: e31–e36. 10.1016/j.forsciint.2008.05.002 18602779

[27] Puschner B, Holstege DM, Lamberski N. Grayanotoxin poisoning in three goats. J Am Vet Med Assoc. 2001; 218: 573–575. doi: 10.2460/javma.2001.218.573 11229512

[28] Uhlig A, Grosche A, Hoops M, Schusser GF. Robinien als Ursache für Vergiftungen beim Pferd. Tierärztl Prax. 2007; 36 (Suppl): 1–5.

[29] Wilkens WM, Cranwell MP. Beechmast poisoning in ponies. Vet Rec. 1990; 127: 435. 2264251

[30] National Centre for Biotechnology Information (NCBI). https://blast.ncbi.nlm.nih.gov/Blast.cgi?CMD=Web&PAGE_TYPE=BlastDocs&DOC_TYPE=FAQ#expect, accessed 23.11.2022.

[31] Remane A. Die Grundlagen des Natürlichen Systems der Vergleichenen Anatomie und der Phylogenetik. Königstein: Koeltz, Königstein; 1971.

[32] Nick P. *Ars comparandi*—molecular convergence versus functional homology. Protoplasma. 2018; 255: 1263–1265. 30120564 10.1007/s00709-018-1301-6

[33] Jürges J, Sahi V, Rios D, Reich E, Bhamra S, Howard C, et al. Product Authenticity versus Globalisation—The Tulsi Case. PloS ONE. 2018; 13: e0207763. doi: 10.1371/journal.pone.0207763 30475878 PMC6261265

[34] Kanbar A, Beisel J, Wetters S, Gutierrez MT, Graeff-Hönninger S, Nick P. A rapid, simple, and reliable assay to authenticate Peruvian kiwicha (A. caudatus) for food applications. Eur J Food Res Technol. 2022; 248: 2779–2797.

[35] Elias SG, Copeland LO, McDonald MB, Baalbaki RZ. Seed Testing: Principles and Practices. Michigan: Michigan State University Press; 2012.

[36] Pompanon F, Deagle BE, Symondson WO, Brown DS, Jarman SN, Taberlet P. Who is eating what: diet assessment using next generation sequencing. Mol Ecol. 2012; 21: 1931–1950. doi: 10.1111/j.1365-294X.2011.05403.x 22171763

[37] An J, Moon JC, Kim JH et al. Development of DNA-based species-specific real-time PCR markers for four berry fruits and their application in commercial berry fruit foods. Appl Biol Chem. 2019; 62: 1–6.

