# Supplementary figures and images for: DNA barcoding as new diagnostic tool to lethal plant poisoning in herbivorous mammals
Source: PLoS One. 2023 Nov 15;18(11):e0292275. doi: 10.1371/journal.pone.0292275 (PMC10650979; doi:10.1371/journal.pone.0292275)

## Slide 1
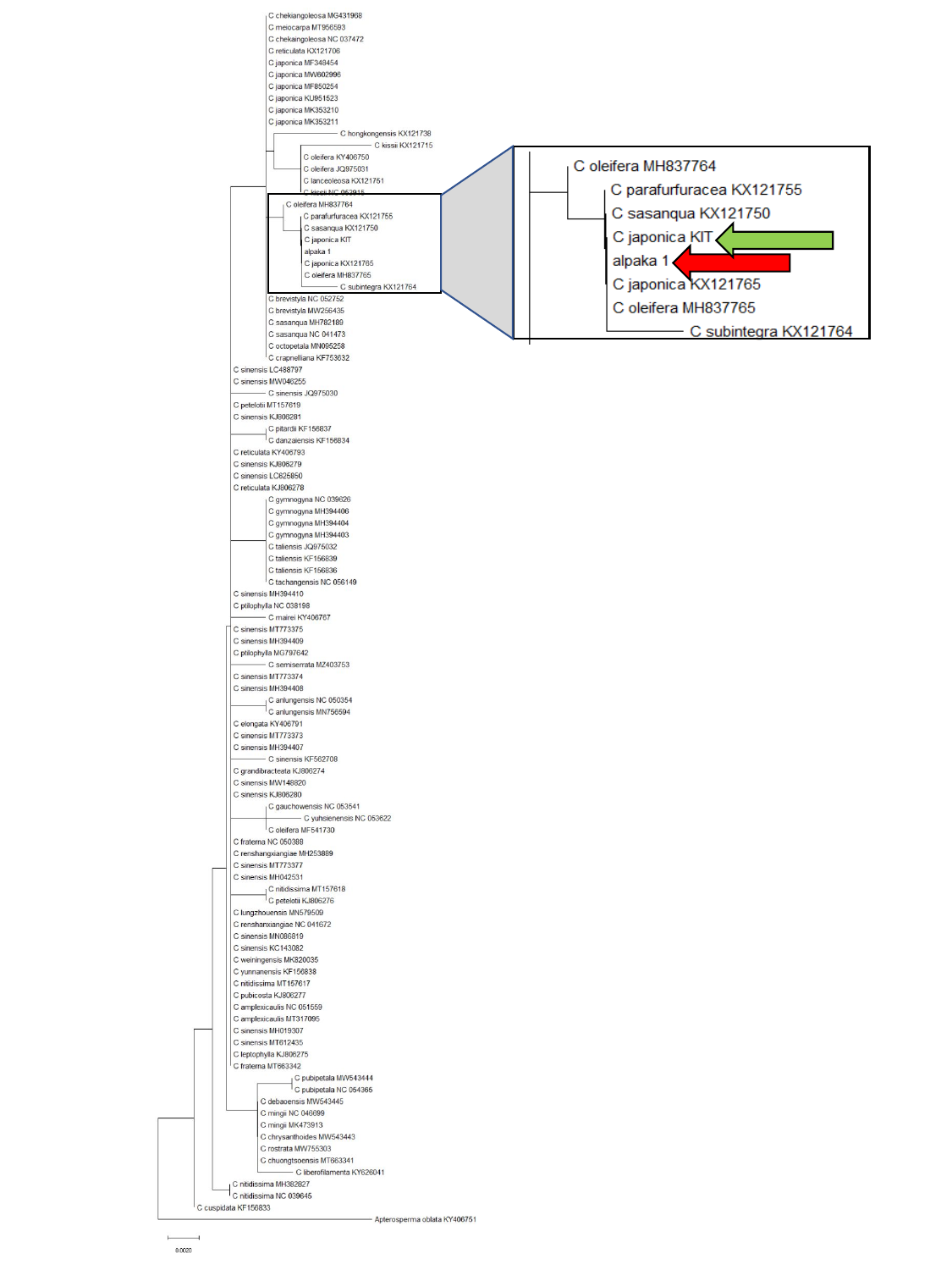

Supplement: S1 Fig — (PPTX) [file pone.0292275.s001.pptx]

## Slide 1
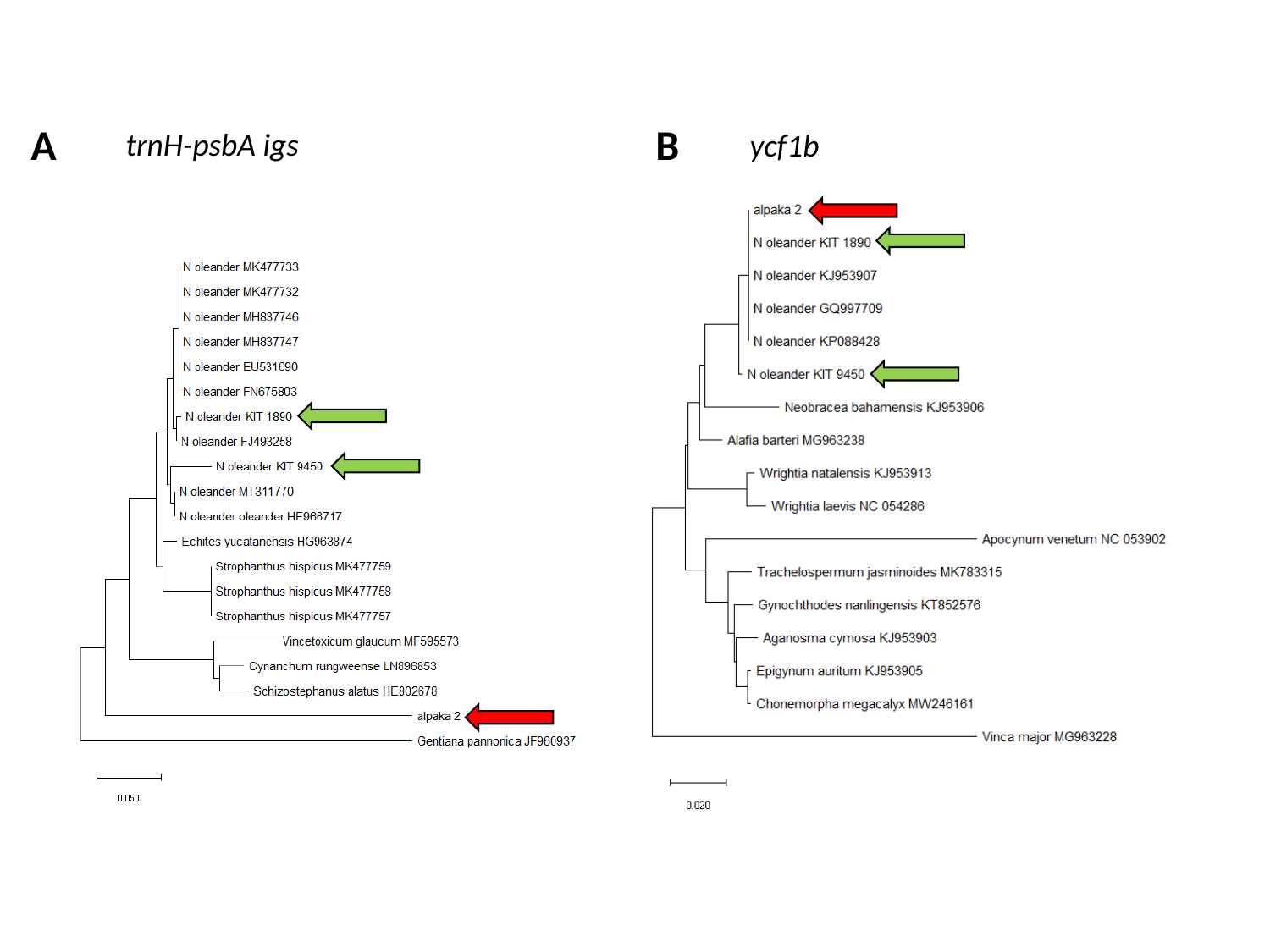

A
B
trnH-psbA igs
ycf1b

Supplement: S2 Fig — (PPTX) [file pone.0292275.s002.pptx]

## Slide 1
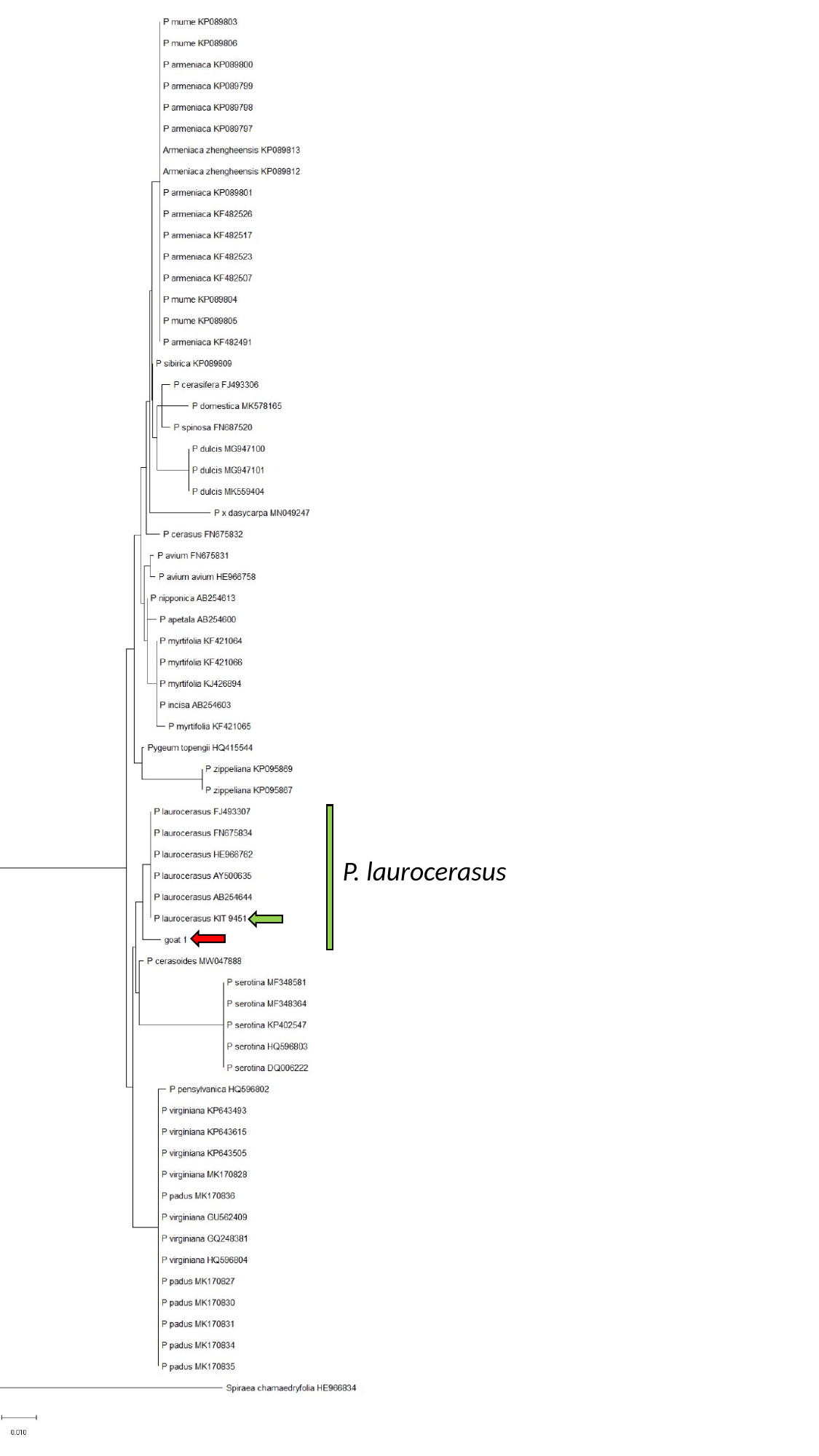

P. laurocerasus

Supplement: S3 Fig — (PPTX) [file pone.0292275.s003.pptx]

## Slide 1
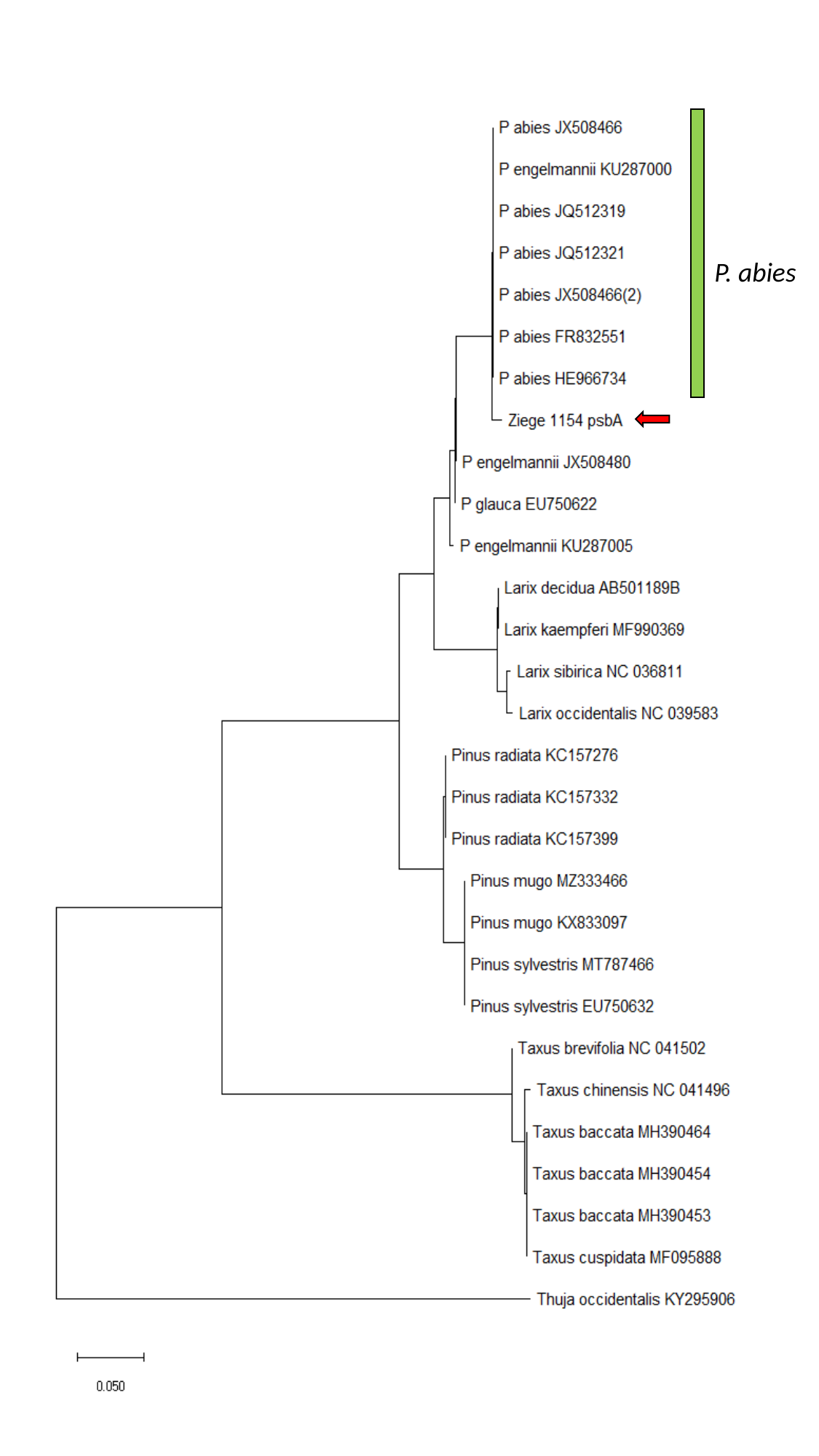

P. abies

Supplement: S4 Fig — (PPTX) [file pone.0292275.s004.pptx]

## Slide 1
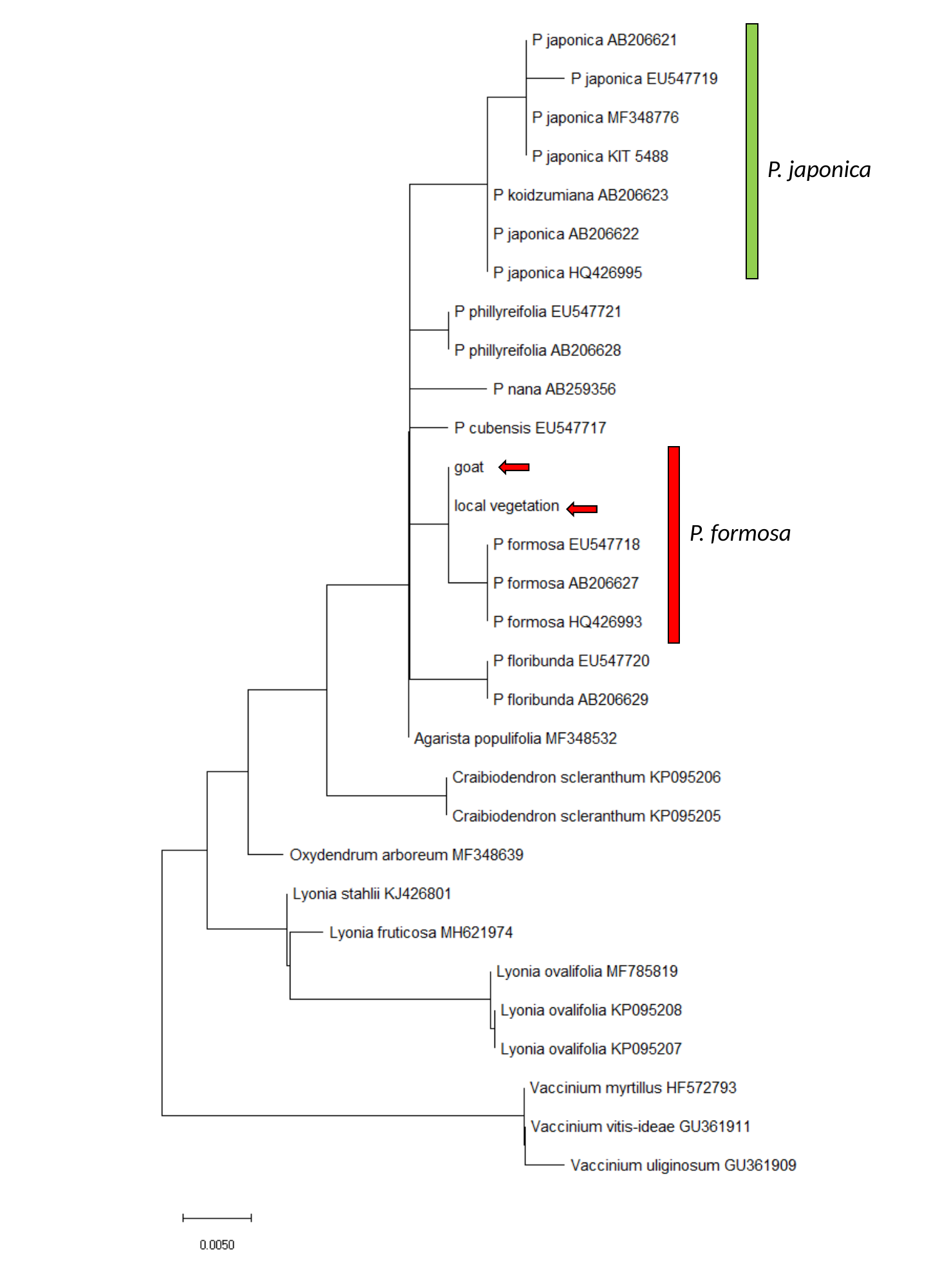

P. japonica
P. formosa

Supplement: S5 Fig — (PPTX) [file pone.0292275.s005.pptx]

## Slide 1
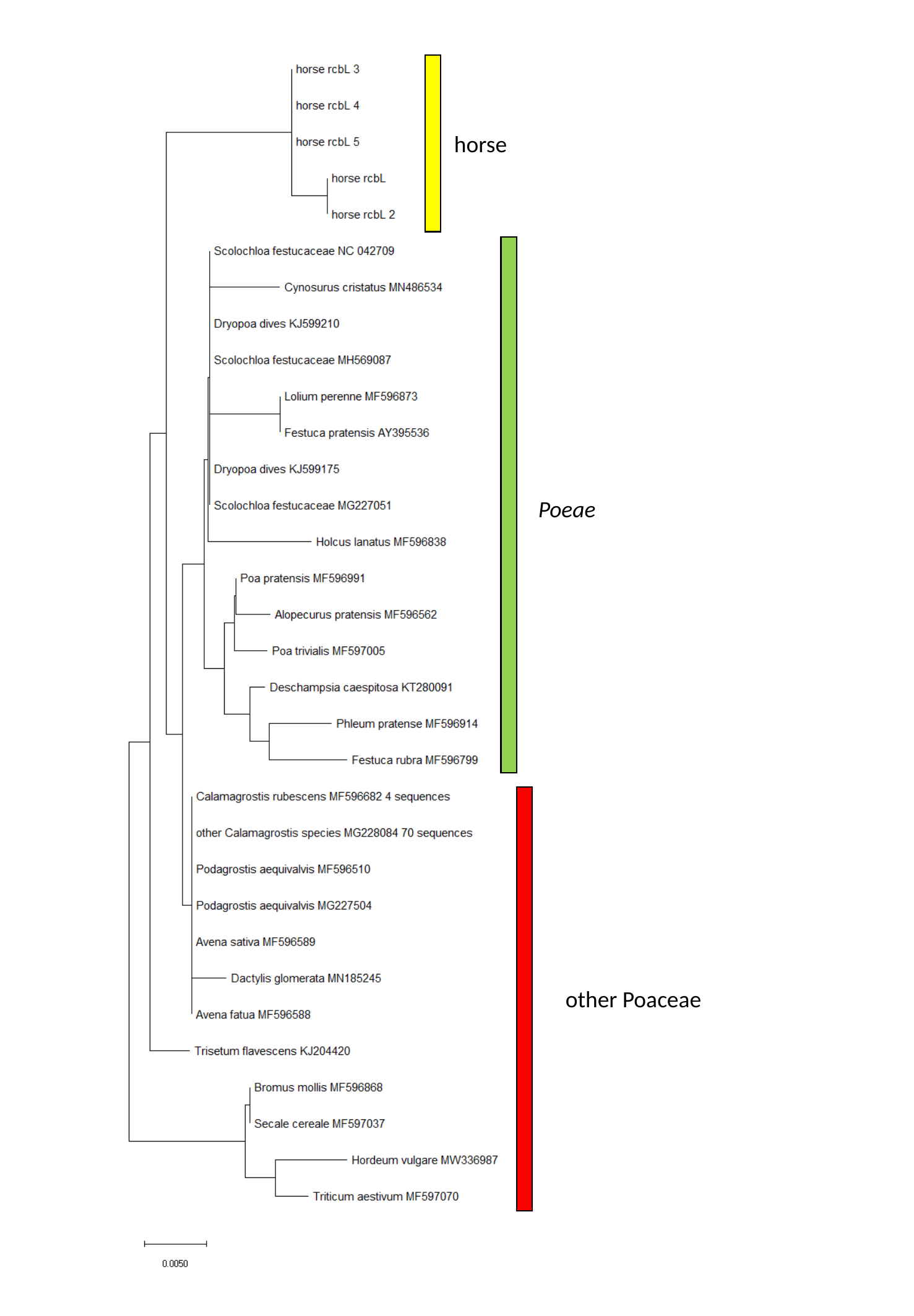

horse
Poeae
other Poaceae

Supplement: S7 Fig — (PPTX) [file pone.0292275.s007.pptx]

## Slide 1
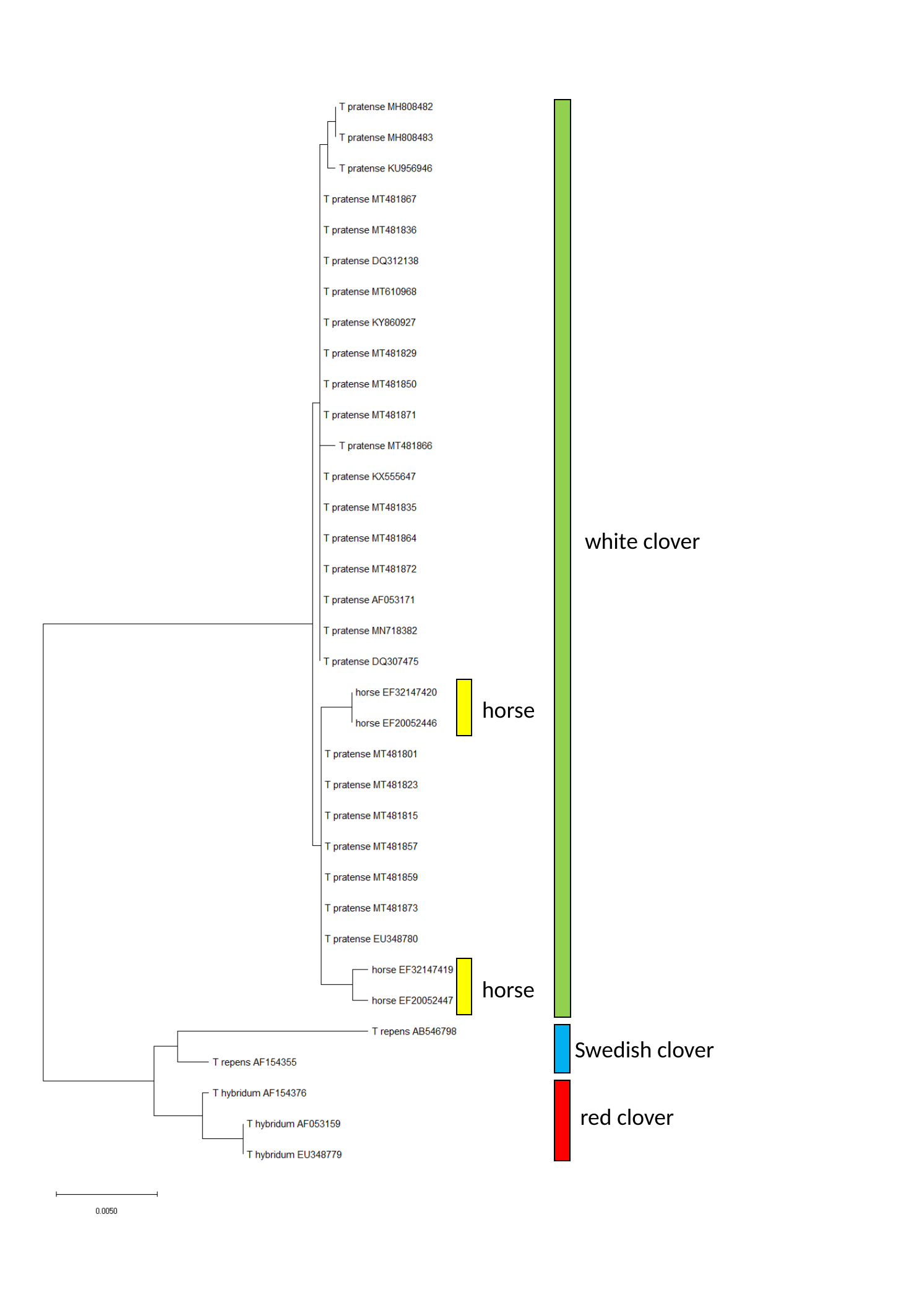

white clover
horse
horse
Swedish clover
red clover

Supplement: S8 Fig — (PPTX) [file pone.0292275.s008.pptx]

## Slide 1
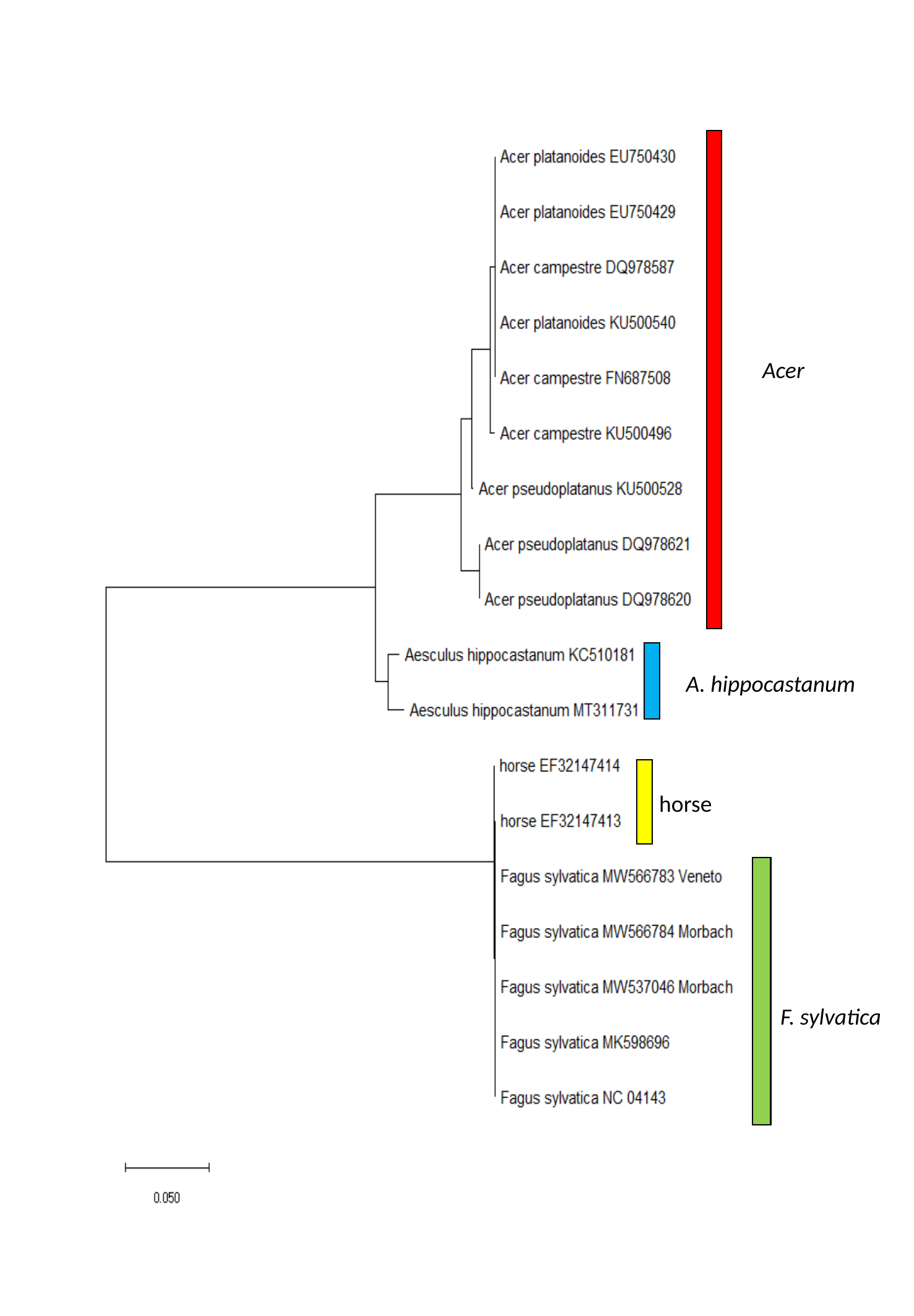

Acer
A. hippocastanum
horse
F. sylvatica

Supplement: S9 Fig — (PPTX) [file pone.0292275.s009.pptx]
