# Supplementary material for: DNA barcoding as new diagnostic tool to lethal plant poisoning in herbivorous mammals
Source: PLoS One. 2023 Nov 15;18(11):e0292275. doi: 10.1371/journal.pone.0292275 (PMC10650979; doi:10.1371/journal.pone.0292275)
Supplement: S6 Fig — (PPTX) [file pone.0292275.s006.pptx]

## Slide 1
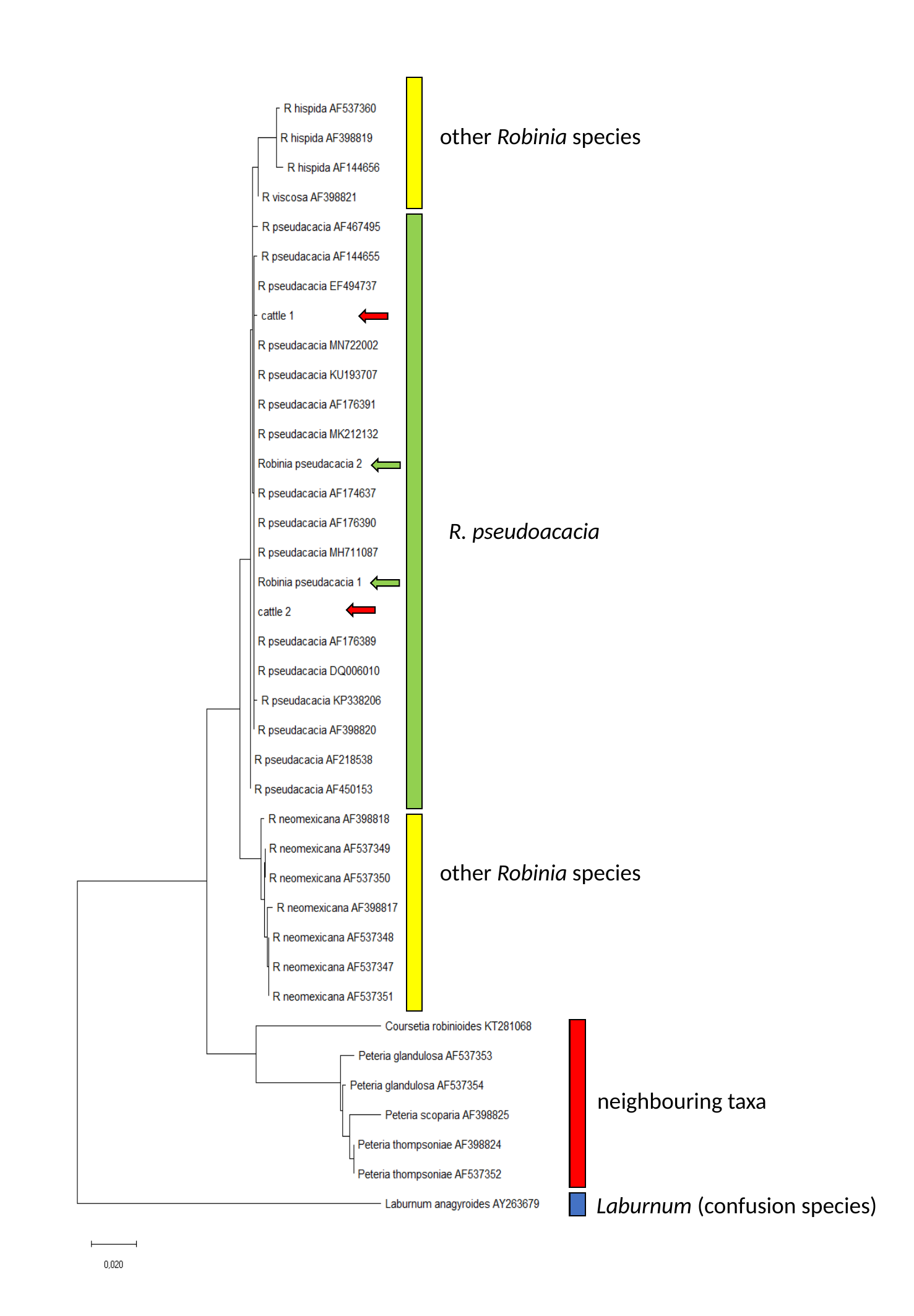

other Robinia species
R. pseudoacacia
other Robinia species
neighbouring taxa
Laburnum (confusion species)
